# A systematic review of the psychometric properties of physical performance tests for sarcopenia in community-dwelling older adults

**DOI:** 10.1093/ageing/afae113

**Published:** 2024-06-08

**Authors:** Sabien H Exter, Niek Koenders, Philip Wees, Manon G A Berg

**Affiliations:** Department of Gastro-enterology and Hepatology, Dietetics and Intestinal Failure, Radboud University Medical Center, Nijmegen, the Netherlands; Department of Rehabilitation, Radboud University Medical Center, Nijmegen, the Netherlands; IQ Healthcare, Radboud University Medical Center, Nijmegen, the Netherlands; Department of Gastro-enterology and Hepatology, Dietetics and Intestinal Failure, Radboud University Medical Center, Nijmegen, the Netherlands

**Keywords:** physical performance, psychometric properties, community-dwelling older adults, validity, reliability, older people, systematic review

## Abstract

**Background:**

This review provides an overview of the psychometric properties of the short physical performance battery (SPPB), timed up and go test (TUG), 4 m gait speed test (4 m GST) and the 400 m walk test (400 m WT) in community-dwelling older adults.

**Methods:**

A systematic search was conducted in MEDLINE, CINAHL and EMBASE, resulting in the inclusion of 50 studies with data from in total 19,266 participants (mean age 63.2–84.3). Data were extracted and properties were given a sufficient or insufficient overall rating following the COSMIN guideline for systematic reviews of patient-reported outcome measures. Quality of evidence (QoE) was rated using the Grading of Recommendations Assessment, Development and Evaluation (GRADE) approach.

**Results:**

The SPPB was evaluated in 12 studies, TUG in 30, 4 m GST in 12 and 400 m WT in 2. Reliability of the SPPB, TUG and 4 m GST was rated sufficient (moderate to good QoE). The measurement error of the SPPB was rated insufficient (low QoE). Criterion validity for the SPPB was insufficient in indicating sarcopenia (moderate QoE), while the TUG was sufficient and insufficient for determining mobility limitations (low QoE) and activities of daily living disability (low QoE), respectively. Construct validity of the SPPB, TUG, 4 m GST and 400 m WT was rated insufficient in many constructs (moderate to high QoE). Responsiveness was rated as insufficient for SPPB (high QoE) and TUG (very low QoE), while 4 m GST was rated as sufficient (high QoE).

**Conclusion:**

Overall, the psychometric quality of commonly used physical performance tests in community-dwelling older adults was generally rated insufficient, except for reliability. These tests are widely used in daily practice and recommended in guidelines; however, users should be cautious when drawing conclusions such as sarcopenia severity and change in physical performance due to limited psychometric quality of the recommended measurement instruments. There is a need for a disease-specific physical performance test for people with sarcopenia.

This research received no specific grant from any funding agency and was registered a priori using the International Prospective Register of Systematic Reviews (PROSPERO) (CRD42022359725).

## Key Points

- The reliability of short physical performance battery, timed up and go test and 4 m gait speed test is sufficient in community-dwelling older adults.
- There is a need for a disease-specific physical performance test for people with sarcopenia.
- Caution is needed when using these tests in sarcopenic patients due to limited psychometric quality.

## Introduction

Ageing is an inevitable process that affects every human being and is associated with a decline in physical performance. Physical performance encompasses whole-body functioning related to muscle strength, balance, flexibility, endurance and mobility [1]. Poor physical performance in older people results in the loss of cognitive function, increased fall risk, decreased quality of life, worse clinical outcomes and a loss of independence in older people’s ability to perform activities of daily living (ADL) [2–4]. Additionally, reduced physical performance indicates the progression and severity of sarcopenia, an age-related skeletal muscle disorder involving the accelerated loss of muscle mass and function [5]. Sarcopenia is related to frailty, especially in terms of low grip strength and low physical performance [6].

As the number of older adults increases and healthcare resources become stretched, it is more crucial than ever for older adults to maintain their independence and promote living at home. This is essential not just for the adults themselves but for society as a whole since the larger group of community-dwelling older people with limited physical abilities often require a significant amount of care to live at home or may need admission to care facilities, leading to increased pressure on care and care costs [7]. Physical performance monitoring seems important to identify adults at risk and evaluate outcomes of treatment, making it vital to have reliable, valid and responsive methods for assessing physical performance [1].

Healthcare professionals and researchers should select physical performance measurement outcomes based on their psychometric properties, which include reliability, validity and responsiveness [8]. Moreover, a healthcare professional may determine physical performance to identify adults at risk that could benefit from interventions to improve their physical performance and overall quality of life. Therefore, a reliable and valid performance test is needed to do this correctly. Researchers may use physical performance outcomes as a part of a clinical trial aimed at improving physical performance and should therefore use a test with high responsiveness to capture change. Additionally, capturing change may also be the goal of healthcare providers to evaluate treatment outcomes by monitoring physical performance. This monitoring of community-dwelling older people several times over extended periods allows healthcare professionals to identify the need for intervention or assess the effects of an intervention over time. More assessment and, thus, earlier intervention could prevent further decline in physical performance and associated adverse health effects [4].

For individuals with sarcopenia, the assessment of physical performance is crucial. It serves as a key parameter indicating the severity of sarcopenia and can be used to initiate and evaluate interventions. There is an abundance of performance tests available to measure physical performance; however, there is no overview of their psychometric qualities. This systematic review provides this overview and focuses on physical performance measurement outcomes as outlined in the European guideline for sarcopenia definition and diagnosis (EWGSOP2) [1, 9]. The physical performance tests reviewed within this paper are the short physical performance battery (SPPB), timed up and go test (TUG) and two types of walking tests: the 4 m gait speed test (4 m GST) and the 400 m walk test (400 m WT) [9].

Given the growing number of community-dwelling older people who aim to live at home for as long as possible, it is crucial to evaluate the psychometric properties of physical performance tests. This review provides an in-depth overview of the psychometric properties of tests used to assess physical performance in the community-dwelling older population.

## Methods

### Protocol and registration

This review was conducted and reported following an adaptation of the COnsensus-based Standards for the selection of health Measurement Instruments (COSMIN) guideline for systematic reviews of patient-reported outcome measures [10]. Adaptations to the original COSMIN guideline have been made for the analysis of performance-based outcome measurement instruments instead of PROMs [11]. This protocol has been registered a priori with PROSPERO (CRD42022359725) and was reported according to an in-development reporting guideline for systematic reviews of outcome measurement instruments by Preferred Reporting Items for Systematic Reviews and Meta-analyses (PRISMA) and COSMIN [12].

### Search strategy

The search strategy has been developed in collaboration with an experienced librarian. Search terms included categories for older people, sarcopenia, community-dwelling, short physical performance battery, timed up and go, gait speed test, walking test and psychometric properties. Search terms used were equal across databases (MEDLINE, CINAHL and EMBASE) although translated into the specific database vocabulary. The full search strategy can be seen in Appendix A. Searches were performed from database inception until 20 September 2022. Results were exported to EndNote for duplicate removal.

### Screening

Deduplicated results were transferred to the systematic review manager Rayyan for the first screening based on title and abstract. A second screening was performed based on full text. Two reviewers independently carried out both the first and second screenings, and any disagreements were discussed and resolved after both rounds of screening.

### Eligibility criteria

Studies reporting on individuals 60 years of age or older who live in the community (not hospitalised or institutionalised) were eligible for inclusion. The selected physical performance tests for this review are commonly used by physical therapists and have previously been selected by EWGSOP2 to determine sarcopenia severity. The EWGSOP2 working group selected these tests based on the feasibility for healthcare providers to use in daily practice and can be performed within many settings as they require no to a very minimal amount of equipment [9]. Therefore, included studies were observational studies and report on the psychometric properties (reliability, validity or responsiveness) of the TUG [13, 14], SPPB [15, 16], 4 m GST or 400 m WT. A description of these tests can be found in Appendix B. Excluded from this review were studies focusing on specific patient groups other than sarcopenia, such as patients with dementia or hip fracture. Additionally, papers reporting on reviews or intervention studies, and those written in a language other than English or Dutch, were excluded.

### Methodological quality

The COSMIN Risk of Bias tool [17] was used to critically appraise studies reporting on reliability and measurement error. The COSMIN Risk of Bias checklist [18] was used to critically appraise studies reporting on validity and responsiveness. This appraisal was completed by two independent reviewers; a consensus meeting was held to resolve conflicts.

### Data extraction

Data extraction was carried out systematically by two independent reviewers. Both reviewers extracted data from all included papers using a data extraction form. The individually extracted data were checked during a consensus meeting. The following data were collected from all included articles: study reference, participant characteristics, outcome measures studied and type of psychometric properties tested.

Participant characteristics including sex, age and body mass index (BMI) were recorded. Additional to physical performance data, data on co-morbidities was recorded when available. Finally, data on all psychometric properties measured for a physical performance test were recorded for analysis.

### Evaluation of psychometric evidence

The psychometric properties evaluated were reliability, validity and responsiveness. Reliability refers to the degree to which a measurement is free from measurement error. This review contains inter-rater, intra-rater and test–retest reliability measures. Additionally, data on measurement errors are recorded. Validity is the degree to which an outcome measure measures the construct it purports to measure. Validity measures within this review contain construct validity and criterion validity. Responsiveness is the ability of an outcome measure to detect change over time in the construct to be measured [19].

An overall rating was given to the physical performance measure based on the criteria for good measurement properties [20]. The overall rating was scored sufficient (+) when the criteria were met, insufficient (−) when they were not met or indeterminate (?) when not enough data were available. Further information on the rating system can be found in Appendix C.

Possible heterogeneity among study results was explored using subgroup analysis. A modified Grading of Recommendations Assessment, Development and Evaluation (GRADE) approach was used to evaluate the quality of evidence available for each outcome measure (high, moderate, low, very low evidence) [10]. Two independent reviewers performed this; a consensus meeting was held to resolve conflicts.

A summary of findings table was prepared to tabulate the results of each psychometric property by measurement outcome and, if relevant, grouped construct.

## Results

**Figure 1:**
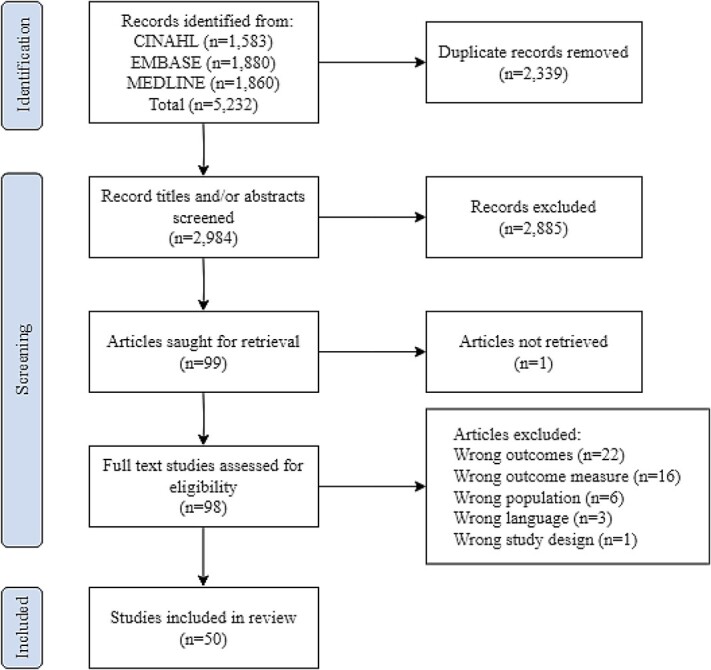


A total of 5,323 records were retrieved from three databases: CINAHL (1583), EMBASE (1880) and MEDLINE (1860). Following title and abstract screening, 98 full-text studies were retrieved and screened against the eligibility criteria. Next, 48 articles were excluded for reasons as mentioned in Figure 1.

### Study characteristics

The 50 included studies were performed in 19 countries (Table 1). All studies were observational. Sample size ranged from 10 to 7,104 participants, and a total of 19,266 participants are presented in this review. The SPPB was researched in 12 studies [21–34], TUG in 30 studies [21, 22, 29, 35–64], 4 m GST in 12 studies [23, 24, 31, 33, 36, 43, 52, 65–69] and the 400 m WT in 2 studies [69, 70]. Inter-rater reliability was analysed in 4 studies [38, 49, 54, 61], intra-rater reliability in 2 studies [38, 61] and test–retest reliability in 9 studies [25, 29, 36, 44, 60, 62, 65, 66, 69]. Measurement error was researched in 6 studies [29, 36, 44, 49, 62, 66], criterion validity in 3 studies [27, 48, 51] and construct validity in 38 studies [21–24, 26, 28–30, 32–35, 37–47, 50–53, 55–59, 64–70]. Lastly, responsiveness was measured in 2 studies [31, 51].

**Table 1:** Study characteristics and demographics. NR, not reported; SD, standard deviation; USA, United States of America

Reference	Participants	Age: mean (SD)	BMI: mean (SD)	Outcome measures	Psychometric properties tested	Country
Alcock [35]	*n* = 39, sex 39 f, 0 m	71.5 (7.3)	NR	TUG	Construct validity	UK
Balachandran [21]	validity *n* = 51, sex 32f, 19 m reliability *n* = 36, sex 19f, 17 m	Validity: 71.3 (5.7) Reliability: 70.4 (5.4)	Validity: 26.6 (5.4) Reliability: 27.4 (5.4)	SPPB TUG	Construct validity	USA
Balasubramanian [22]	*n* = 40, sex 26 f, 14 m	73.3 (6.9)	NR	SPPB TUG	Construct validity	USA
Bean [23]	*n* = 138, sex 95 f, 43 m	75.4 (6.9)	27.5 (NR)	SPPB 4 m GST	Construct validity	USA
Beauchamp [36]	*n* = 147, sex 71 f, 76 m	69 (10)	28.6 (5.3)	TUG 4 m GST	Test–retest reliability Measurement error	Canada
Cho [37]	*n* = 167, sex NR	78 (7)	NR	TUG	Construct validity	USA
Creel [38]	*n* = 30, sex 17 f, 13 m	77.5 (7)	NR	TUG	Construct validity Inter-rater reliability Intra-rater reliability	USA
de Vreede [39]	Validation *n* = 24, sex 24 f, 0 m	Validation 74.6 (4.8)	NR	TUG	Construct validity	Netherlands
Di Fabio [40]	*n* = 35, sex 31 f, 4 m	79.9 (8.5)	NR	TUG	Construct validity	USA
Fernández-Huerta [65]	*n* = 136, sex 103 f, 33 m	72.8 (5.9)	NR	4 m GST	Construct validity Test–retest reliability	Chile
Fusco [24]	*n* = 73, sex 38 f, 35 m	77.6 (8.3)	NR	SPPB 4 m GST	Construct validity	Italy
Gamerman [41]	*n* = 78, sex 65 f, 13 m	76.6 (6.5)	NR	TUG	Construct validity	Israel
Goldberg [66]	intermediate gait *n* = 15, sex 11 f, 4 m fast gait *n* = 15, sex 13 f, 2 m	Intermediate: 74.2 (6.1) Fast: 72.1 (6)	Intermediate: 29.6 (8.6) Fast: 26.2 (4.6)	4 m GST	Test–retest reliability Measurement error Construct validity	USA
Goldberg [42]	*n* = 35, sex 28 f, 7 m	72.8 (1)	28.8 (1.1)	TUG	Construct validity	USA
Gómez [25]	*n* = 150, sex 77 f, 73 m	69.5 (3.1)	NR	SPPB	Test–retest reliability	Colombia
Gordt [43]	*n* = 51, sex 39 f, 12 m	69.9 (7.1)	NR	TUG 4 m GST	Construct validity	Germany
Grey [26]	*n* = 57, sex 41 f, 16 m	f 78.2 (6.4) m 78.2 (7.3)	NR	SPPB	Construct validity	USA
Griswold [44]	*n* = 40, gender 22 f, 18 m	68.7 (8.6)	NR	TUG	Test–retest reliability Measurement error Construct validity	USA
Hachiya [45]	*n* = 31, sex 19 f, 12 m	75.7 (6)	NR	TUG	Construct validity	Japan
Härdi [46]	*n* = 109, sex 91 f, 18 m	74.1 (6.4)	26.3 (4.7)	TUG	Construct validity	Switzerland
Hashidate [47]	*n* = 20, sex 12 f, 8 m	76.6 (5.1)	22.1 (3.2)	TUG	Construct validity	Japan
Kim [48]	*n* = 433, sex NR	73.2 (5.7)	NR	TUG	Criterion validity Construct validity	South Korea
Kristensen [49]	outpatient *n* = 11, sex 7 f, 4 m	Outpatient: 79.2 (6.8)	NR	TUG	Inter-rater reliability Measurement error	Denmark
Kwan [50]	*n* = 280, sex 120 f, 160 m	74.9 (6.4)	NR	TUG	Construct validity	Taiwan
Lee [27]	total *n* = 538, sex 311 f, 227 m 60+ *n* = 299, sex 168 f, 131 m	Total: 59 (19) 61–65: 63 (1); 66–70: 68 (1); 71–75: 73 (2); 76–80: 78 (1); 80+: 84 (2)	Total: 25.2 (4.9) 61–65: 24.0 (2.9); 66–70: 24.0 (3.4); 71–75: 24.2 (3.2); 76–80: 23.7 (3.0); 80+: 23.5 (4.1)	SPPB	Criterion validity	Singapore
Lin [51]	*n* = 1,200, sex 491 f, 709 m	73.4 (NR)	NR	TUG	Criterion validity Construct validity Responsiveness	Taiwan
Looijaard [52]	*n* = 140, sex 81 f, 59 m	80.9 (7.1)	NR	TUG 4 m GST	Construct validity	Netherlands
Löppönen [28]	*n* = 479, sex 287 f, 192 m	NR: 75–85	NR	SPPB	Construct validity	Finland
Maggio [67]	*n* = 172, sex 103 f, 69 m	f 78.2 (5.6) m 79.0 (4.9)	f 26.6 (3.2) m 26.2 (3.8)	4 m GST	Construct validity	Italy
Mathis [29]	*n* = 31, sex 20 f, 11 m	81.1 (8.3)	NR	SPPB TUG	Construct validity Test–retest reliability Measurement error	USA
Minematsu [53]	*n* = 599, sex 340 f, 249 m	f 73.0 (5.2) m 73.7 (5.3)	NR	TUG	Construct validity	Japan
Nepal [54]	*n* = 100, sex 54 f, 46 m	69.1 (8.0)	NR	TUG	Inter-rater reliability	Nepal
Ni [30]	*n* = 50, sex 26 f, 24 m	77.2 (6.1)	NR	SPPB	Construct validity	UK
O’Hoski [55]	*n* = 79, sex NR	68.7 (10.6)	25.5 (4)	TUG	Construct validity	Canada
Olivares [56]	*n* = 7,104, sex 6,243 f, 861 m	NR: 50+	NR	TUG	Construct validity	Turkey
Özden [57]	*n* = 65, sex 34 f, 21 m	68.9 (3.7)	28.1 (4.2)	TUG	Construct validity	Turkey
Pasma [68]	*n* = 288, sex 187 f, 101 m	82.2 (7.1)	25.9 (4.5)	4 m GST	Construct validity	Netherlands
Perera [31]	PEP study *n* = 492, sex 213 f, 279 m	PEP: 74.1 (5.7)	NR	SPPB 4 m GST	Responsiveness	USA
Portegijs [28, 32]	total *n* = 848, sex 526 f, 322 m light PA *n* = 306, sex 218 f, 88 m moderate PA *n* = 253, sex 156 f, 107 m regular PA *n* = 289, sex 152 f, 137 m	total: NR light: 82.5 (7.2) moderate: 80.0 (7.7) regular: 78.7 (5.9)	NR	SPPB	Construct validity	Finland
Riwniak [33]	*n* = 89, sex 57 f, 32 m	74.9 (6.7)	27.4 (5)	SPPB 4 m GST	Construct validity	USA
Rolland [69]	total *n* = 60, sex 53 f, 7 m able to perform both 400 m *n* = 41, sex 36 f, 5 m	total: 84.3 (6.3) able to perform both 400 m: 63.2 (6.4)	NR	4 m GST 400 m WT	Construct validity Test–retest reliability	USA
Schaubert [58]	*n* = 10, sex 2 f, 8 m	74.5 (5.8)	NR	TUG	Construct validity	USA
Schepens [59]	*n* = 35, sex 28 f, 7 m	72.9 (1.1)	28.8 (1.1)	TUG	Construct validity	USA
Simonsick [70]	*n* = 3,075, sex NR	NR	NR	400 m WT	Construct validity	USA
Stanziano [34]	*n* = 145, sex 72 f, 70 m	79.6 (7.2)	NR	SPPB	Construct validity	USA
Steffen [60]	*n* = 96, sex 59 f, 37 m	f: 73 (8) m: 73 (8)	f: 29 (6) m: 28 (5)	TUG	Test–retest reliability	USA
Suwannarat [61]	*n* = 309, sex 193 f, 116 m	Walking device users: 76.6 (5.7) Non-device users: 73 (5.4)	Walking device users: 23.3 (4.1) Non-device users: 22.8 (3.7)	TUG	Inter-rater reliability Intra-rater reliability	Thailand
Suzuki [62]	*n* = 718, sex 521 f, 197 m	f: 71.2 (4.5) m: 73.4 (5.3)	f: 22.1 (3.1) m: 22.8 (2.5)	TUG	Test–retest reliability Measurement error	Japan
Wang [63]	*n* = 268, sex 119 f, 149 m	73.8 (5.2)	NR	TUG	Construct validity	Taiwan
Wrisley [64]	*n* = 35, sex 18 f, 17 m	72.9 (7.8)	NR	TUG	Construct validity	USA

### Participant characteristics

The mean age of participants ranged from 63.2 to 84.3. Mean BMI was reported in 17 studies and ranged 22.1–29.6. Most studies were performed in both sexes, with two studies reporting only female participants [35, 39]. Co-morbidities were reported in various studies and include diabetes [21, 36, 63], gastrointestinal disease [49], heart conditions [21, 63], hypertension [21, 36], mental illness [36], musculoskeletal disease [23, 36], neurologic conditions [36, 40], orthopaedic conditions [40], pulmonary disease [49], respiratory disease [36] and vision disease [36, 63].

Only 2 out of 50 included studies described sarcopenia incidence in their population. Lee *et al.* included between 66 (21.8%) and 71 (23.4%) patients with sarcopenia in a total of 303 participants, depending on the definition used [27]. Out of a total of 140 participants, Looijaard *et al.* described between 5 (3.6%) and 33 (23.6%) to be sarcopenic [52].

### Summary of findings

#### Physical performance average test scores

Mean SPPB score was reported in 16 studies and ranged from 7.88; 11.49. Mean TUG time was reported in 34 studies and ranged from 5.80; 24.10 seconds. Mean 4 m GST was reported in 10 studies and ranged from 0.72; 1.57 m/s. Median time to complete the 400 m WT was reported in one study and ranged from 5.09; 5.36 minutes. Full results of the physical performance test scores can be found in Appendix D.

#### Reliability

Inter-, intra- and test–retest reliability was measured using ICC. Inter-rater reliability of the TUG ranged from 0.81; 0.98, intra-rater reliability of the TUG ranged from 0.96; 0.99 and test–retest reliability of the SPPB, TUG and 4 m GST ranged from 0.64; 0.97. The quality of evidence was deemed to be between moderate and high (Table 2).

**Table 2:** Summary of findings: reliability, measurement error and criterion validity. The overall rating is scored sufficient (+), insufficient (−) or indeterminate (?) when not enough data are available, according to the criteria for good measurement properties [20]. A modified GRADE approach was used to evaluate the quality of evidence [10]. AUC, area under the curve; ICC, intra-class correlation coefficient; SEM, standard error of the mean; MDC, minimal detectable change; N/A, not applicable

	Summarised result: range	Number of participants (number of studies)	Overall rating	Quality of evidence
Reliability				
SPPB	Test–retest reliability (ICC): 0.87;0.93 [25, 29]	181 (2)	+	⊕ ⊕ ⊕◯ Moderate
TUG	Inter-rater reliability (ICC): 0.81;0.98 [38, 49, 54, 61]	450 (4)	+	⊕ ⊕ ⊕ ⊕ High
	Intra-rater reliability (ICC): 0.96;0.99 [38, 61]	339 (2)	+	
	Test–retest reliability (ICC): 0.80;0.97 [29, 36, 44, 60, 62]	1032 (5)	+	
4 m GST	Test–retest reliability (ICC): 0.64;0.97 [36, 65, 66, 69]	373 (4)	+	⊕ ⊕ ⊕◯ Moderate
400 m WT	N/A	N/A	N/A	N/A
Measurement error				
SPPB	SEM: 0.60 [29] MDC_95_: 1.9 [29]	31 (1) 31 (1)	−	⊕◯◯◯ Very low
TUG	SEM: 0.32;0.97 [29, 36, 44, 49, 62] MDC_90_: 2.26 [36] MDC_95_: 1.6;1.8 [29, 49]	947 (5) 31 (1) 42 (2)	?	⊕ ⊕ ⊕ ⊕ High
4 m GST	SEM: 0.05;0.10 [36, 66] MDC_90_: 0.23 [36] MDC_95_: 0.108;0.144 [66]	177 (2) 31 (1) 30 (1)	?	⊕ ⊕ ⊕◯ Moderate
400 m WT	N/A	N/A	N/A	N/A
Criterion validity				
SPPB	Sarcopenia diagnosis (AUC): 0.54;0.65 [27]	538 (1)	−	⊕ ⊕ ⊕◯ Moderate
TUG	Mobility limitations (AUC): 0.72;0.80 [48] ADL disability (AUC): 0.648 [51]	433 (1) 1200 (1)	+ -	⊕ ⊕ ◯◯ Low
4 m GST	N/A	N/A	N/A	N/A
400 m WT	N/A	N/A	N/A	N/A

Measurement error was estimated in the SPPB, TUG and 4 m GST using SEM and ranged from 0.05; 0.97 (Table 2). Quality of evidence for these analyses was between very low and high. The very low quality of evidence was due to serious risk of bias and very serious imprecision in SPPB studies.

#### Validity

Criterion validity was reported for the SPPB, and TUG using area AUC and ranged from 0.54; 0.80 (Table 2). The quality of evidence was low to moderate; studies researching TUG showed very serious risk of bias.

Construct validity was reported in the SPPB, TUG, 4 m GST and 400 m WT using several methods including SRCC, RCC, ICC, linear regression, odds ratio (OR), mean difference and limits of agreement (LoAs). Comparator instruments used to assess construct validity were grouped with similar instruments to make the synthesis of results possible and explore possible heterogeneous results. For example, a group of short walking tests contains, among others, 3, 4 and 6 m walking tests. All groups, as well as the ungrouped results, can be found in Appendixes G and H. Correlation coefficient results ranged from −0.910; 0.93 (Table 3). The quality of evidence ranged between moderate and high.

**Table 3:** Summary of findings: Construct validity. The overall rating is scored sufficient (+), insufficient (−) or indeterminate (?) when not enough data are available, according to the criteria for good measurement properties [20]. Hypotheses used for rating the correlation coefficient: same construct (>0.7), related construct (0.4–0.7) and unrelated construct (<0.4). A modified GRADE approach was used to evaluate the quality of evidence [10]. ICC, intra-class correlation coefficient; PCC, Pearson correlation coefficient; SRCC, Spearman’s rank correlation coefficient

	Summarised result per comparator group: range	Number of participants (number of studies)	Hypothesis used	Overall rating	Quality of evidence
Construct validity					
SPPB	Balance (PCC): −0.700;0.307 [26, 34]	202 (2)	Related construct	−	⊕ ⊕ ⊕◯ Moderate
	Chair stand tests (PCC): 0.410;0.690 [21, 26]	108 (2)	Related construct	+	
	Rest: Free living sit to stand (SRCC): 0.170 [28]	479 (1)	Unrelated construct	+	
	Muscle strength (PCC): 0.190;0.290 [21, 30] Muscle strength (SRCC): 0.420;0.510 [23]	101 (2) 138 (1)	Unrelated construct	+ -	
	Physical functioning questionnaires (PCC): 0.290;0.370 [29, 33] Physical functioning questionnaires (SRCC): 0.290;0.750 [22, 24, 32]	120 (2) 961 (3)	Related construct	- -	
	Short walking test (PCC): 0.190;0.350 [26] Short walking test (SRCC): 0.776 [24] Long walking test (PCC): 0.630 [26]	57 (1) 73 (1) 57 (1)	Related construct	-—+	
TUG	Balance (PCC): −0.690;0.680 [42, 44, 55, 59] Balance (SRCC): −0.679;0.881 [37, 41, 57] Balance (NR): −0.550 [51]	199 (4) 310 (3) 1,200 (1)	Related construct	- + ?	⊕ ⊕ ⊕ ⊕ High
	Chair stand tests (PCC): 0.370;0.918 [21, 35, 58]	100 (3)	Related construct	−	
	Mobility (PCC): 0.790;0.89 [38] Mobility (SRCC): −0.651 [37]	30 (1) 167 (1)	Related construct	- +	
	Muscle strength (PCC): −0.290;-0.228 [21, 56] Muscle strength (partial regression coefficient): −0.671;-0.079 [53]	7,155 (2) 299 (1)	Unrelated construct	+ -	
	Physical functioning questionnaires (PCC): −0.910;0.210 [29, 35, 39, 46, 50] Physical functioning questionnaires (SRCC): −0.740;0.495 [21, 37, 40, 43, 47] Physical functioning questionnaires (NR): −0.450 [51]	472 (5) 313 (5) 1,200 (1)	Related construct	- — ?	
	Sarcopenia (OR): 1.00;1.01 [52]	140 (1)	Unrelated construct	?	
	Short walking test (PCC): −0.830;0.960 [35, 45] Short walking test (SRCC) -0.840;0.596 [57, 64] Short walking test (NR): −0.530 [51] Long walking test (PCC): −0.573 [56] Long walking test (SRCC): −0.752 [37]	70 (2) 100 (2) 1,200 (1) 7,104 (1) 164 (1)	Related construct	-—? + -	
	Rest (PCC): −0.300;0.317 [50, 56] Rest (SRCC): −0.020;0.370 [40]	7,284 (2) 35 (1)	Unrelated construct	+ +	
4 m GST	Balance (PCC): 0.650 [43, 66]	30 (1)	Unrelated construct	−	⊕ ⊕ ⊕◯ Moderate
	Muscle strength (PCC): 0.380;0.510 [67] Muscle strength (SRCC): 0.290;0.560 [23]	172 (1) 138 (1)	Unrelated construct	- -	
	Physical functioning questionnaires (PCC): 0.570 [33] Physical functioning questionnaires (SRCC): −0.580;0.617 [24, 43]	89 (1) 124 (2)	Unrelated construct	- -	
	Sarcopenia (OR): 0.410;1.540 [52]	140 (1)	Unrelated construct	?	
	Short walking test (PCC): 0.824 [69] Short walking test (SRCC): 0.930 [69] Short walking test (ICC): 0.867 [65] Short walking test (mean difference^a^): −0.110 [68] Short walking test (LoA^a^): −0.13;0.10 [68] Long walking test (PCC): 0.490;0.590 [67] Long walking test (mean difference^b^): −0.030 [68] Long walking test (LoA^b^): −0.08; 0.03 [68]	60 (1) 60 (1) 136 (1) 288 (1) 288 (1) 172 (1) 288 (1) 288 (1)	Related construct Related construct	- - - ? ? + ? ?	
400 m WT	Balance (PCC): −0.531; −0.307 [70]	3,075 (1)	Unrelated construct	−	⊕ ⊕ ⊕◯ Moderate
	Chair stand (PCC): −0.416; −0.376 [70]	3,075 (1)	Unrelated construct	−	
	Physical functioning questionnaires (PCC): −0.614; −0.238 [70]	3,075 (1)	Unrelated construct	−	
	Short walking test (PCC): −0.838; −0.589 [70] Short walking test (SRCC): 0.930 [69]	3,075 (1) 60 (1)	Related construct	- -	

^a^Compared to 10 m walking test

^b^Compared to 6 minute walking test

#### Responsiveness

Responsiveness was reported in the SPPB, TUG and 4 m GST by AUC or SEM, small meaningful change and substantial meaningful change. The outcomes ranged from 0.05; 1.42 (Table 4). The quality of evidence for these findings was between very low and high with serious indirectness and extremely serious risk of bias in the assessment of the TUG.

**Table 4:** Summary of findings: Responsiveness. The overall rating is scored sufficient (+), insufficient (−) or indeterminate (?) when not enough data are available, according to the criteria for good measurement properties [20]. A modified GRADE approach was used to evaluate the quality of evidence [10]. AUC, area under the curve; N/A, not applicable; SEM, standard error of the mean

	Summarised result	Number of participants (number of studies)	Overall rating	Quality of evidence
Responsiveness				
SPPB	Small meaningful change: 0.54 [31] Substantial meaningful change: 1.34 [31] SEM meaningful change: 1.42 [31]	429 (1)	−	⊕ ⊕ ⊕ ⊕ High
TUG	AUC: 0.592 [51]	1200 (1)	−	⊕◯◯◯ Very low
4 m GST	Small meaningful change: 0.05 [31] Substantial meaningful change: 0.12 [31] SEM meaningful change: 0.06 [31]	429 (1)	+	⊕ ⊕ ⊕ ⊕ High
400 m WT	N/A	N/A	N/A	N/A

The risk of bias assessment and full GRADE scorings can be found in Appendixes E and F.

## Discussion

This systematic review provides an overview of the psychometric properties of the SPPB, TUG, 4 m GST and 400 m WT in community-dwelling older adults. Reliability of the SPPB, TUG and 4 m GST was rated sufficient (moderate to good quality of evidence). The measurement error of the SPPB was rated insufficient (low quality of evidence) and indeterminate of the TUG and 4 m GST (respectively, high and moderate quality of evidence). Criterion validity of the SPBB was rated insufficient for diagnosing sarcopenia (moderate quality of evidence). The psychometric quality of the TUG was rated sufficient for determining mobility limitations (low quality of evidence), though insufficient for ADL disability (low quality of evidence). The construct validity of the SPPB, TUG, 4 m GST and 400 m WT was rated mainly insufficient (moderate to high quality of evidence). Responsiveness of the SPPB and TUG was rated insufficient (respectively, high quality and very low quality of evidence and 4 m GST sufficient (high quality of evidence).

A previous review showed good to excellent test–retest reliability of the SPPB (0.82–0.92) in older adults in multiple settings [71]. Yet another review showed excellent test–retest (0.96–0.97), inter-rater (0.99) and intra-rater (0.94–0.99) reliability of the TUG in typical adults [72]. Our current review also rated sufficient reliability for these psychometric properties, although we focused on community-dwelling older adults. The review of Freiburger *et al.* [73] also examined validity and responsiveness of the SPPB in community-dwelling older adults and rated the psychometric quality as ‘very good’ and ‘good’. However, the authors used different quality criteria for overall rating of the psychometric quality. A different review assessing the validity and reliability of performance tests used in community-dwelling older people examined physical performance tests and other measures used in sarcopenia screening [74]. In the current review, the evidence of SPPB on construct validity with related comparators was mainly rated insufficient as the hypotheses were not confirmed. These outcomes conflict with the findings of a previous systematic review that showed sufficient construct validity of the SPPB based on PCC results for 400 m WT and mobility disability [74]. No additional measurement for mobility disability was performed in the study referenced; however, 400 m WT results were interpreted as mobility indicator [75]. The discrepancy in the overall sufficiency rating can be explained by a difference in hypotheses used to test construct validity. In the current study, the construct validity of the SPPB compared to short walking tests, like the 400 m WT, using PCC is tested on the hypothesis of 0.4 ≤ PCC ≤ 0.7 for related constructs. The result in this review shows PCC = 0.776 and does not align with the hypothesis. This result, however, does align with the hypothesis in the previous review of PCC ≥ 0.50 [74].

It should be acknowledged that the SPPB contains a short walking test, which influences the construct validity when using short walking tests as a comparator. The lack of a gold standard to measure physical performance makes it hard to test criterion validity, as this is part of the definition of criterion validity [19]. However, the TUG seems valid to determine mobility limitations with a low quality of evidence. Other reviews do not report on the criterion validity of physical performance outcome measures. To be able to indicate sarcopenia severity, a test needs sufficient criterion validity or construct validity. The validity varies by the definition of sarcopenia used. For the validity of the SPPB, the study by Lee *et al.* used the Asian Working Group for Sarcopenia 2019 definition [27]. Looijaard *et al.* split their results for the validity of the TUG according to five different definitions of sarcopenia according to Baumgartner, EWGSOP, Foundation for the National Institutes of Health, International Working Group on Sarcopenia and Janssen [52]. Responsiveness was rated insufficient in the SPPB (high quality of evidence). This conclusion is in line with a previous review [71].

This systematic review has several strengths. The systematic search strategy was developed with information specialists, ensuring a thorough search of the available literature. The search resulted in the inclusion of 50 studies that offer a comprehensive and broad overview of the psychometric properties that have been examined. Additionally, this systematic review was established using a state-of-the-art methodology, which allows for an unbiased overall rating and grading of evidence [10]. The thorough methodological approach ensures the objective evaluation of psychometric properties with minimised bias. The grading of evidence using the GRADE approach provides a structured and transparent overview of the quality of evidence. Lastly, a significant strength lies in the alignment of the scope of this review with existing guidelines [9]. By critically examining and appraising the outcome measures recommended in the current guidelines, this review can offer insights for later revisions. One important insight is that the physical performance measures recommended have yet to be exhaustively approved on reliability, validity and responsiveness. Therefore, caution must be taken with the interpretation of test results. There are also some limitations to this systematic review. There is a large difference in the quantity of available evidence about psychometric properties of different performance tests. This overview shows no measurement error of the TUG and 4 m GST due to missing data on their M(C)IC. No criterion validity could be reported for the 4 m GST. No reliability, measurement error, criterion validity or responsiveness of 400 m WT has been reported in community-dwelling older adults. Furthermore, due to the considerable heterogeneity between studies in design, data analysis and reporting, it was not possible to perform a meta-analysis, and results had to be narratively summarised. [9].

Although the measurement outcomes discussed are suggested to assess sarcopenia severity as stated by EWGSOP2 [9], this systematic review could only include two studies that showed data on patients diagnosed with sarcopenia [27, 52]. A maximum of 22.8% [27] and 23.6% [52] of participants had sarcopenia, according to different sarcopenia definitions that influence prevalence, highlighting the paucity of research on the psychometric properties of physical performance tests in this population. This review, therefore, indicates the need for more studies reporting on the psychometric properties of physical performance outcome measures in the sarcopenic population because it is unclear whether results on community-dwelling older adults can be generalised to the sarcopenic population. Such studies are necessary to verify the tests’ reliability, validity and responsiveness in patients with sarcopenia.

Reliable and valid measurement performance tests are required to indicate patients (at risk) for limited physical performance or for evaluating interventions. This review shows the SPPB, TUG and 4 m GST are reliable instruments. The SPPB has insufficient reliability according to measurement error and has insufficient criterion validity to indicate sarcopenia. The criterion validity of TUG was sufficient and insufficient in determining mobility limitations and ADL disability, respectively. Construct validity was often rated insufficient for every measurement outcome. The 4 m GST has sufficient responsiveness to analyse changes in physical performance in community-dwelling older adults. The quality of evidence varied from very low to high and was mostly moderate. The psychometric quality of commonly used physical performance tests in community-dwelling older adults seems generally insufficient, except for reliability. The SPPB, TUG, 4 m GST and 400 m WT are widely used in daily practice and recommended in clinical guidelines; however, users should be cautious when drawing conclusions such as sarcopenia severity and change in physical performance due to limited psychometric quality of the recommended measurement instruments. There is a need for a disease-specific physical performance test for people with sarcopenia.

## Supplementary Material

aa-23-1095-File002_afae113
