# Supplementary material for: A systematic review of the psychometric properties of physical performance tests for sarcopenia in community-dwelling older adults
Source: Age Ageing. 2024 Jun 8;53(6):afae113. doi: 10.1093/ageing/afae113 (PMC11162262; doi:10.1093/ageing/afae113)
Supplement: aa-23-1095-File002_afae113 [file aa-23-1095-file002_afae113.docx]

Supplementary file

**Appendix A: Full search strategy**

**Appendix B: Description of instruments**

**Appendix C: Overall rating methodology**

**Appendix D: Mean values of physical performance tests**

**Appendix E: Risk of Bias Assessment**

**Appendix F: GRADE Evaluation**

**Appendix G: Construct validity comparator groups**

# Appendix H: Construct validity results

# Appendix I: PRISMA Checklist

# Supplementary File References

**Appendix A: Full search strategy**

**PubMed**

**Older people**

"aged"[MeSH Terms] OR

"aging"[MeSH Terms] OR

"geriatrics"[MeSH Terms] OR

"Frail Elderly"[Mesh] OR

"Sarcopenia"[MeSH Terms] OR

"aged"[Title/Abstract] OR

"geriatric*"[Title/Abstract] OR

"elder*"[Title/Abstract] OR

"ageing"[Title/Abstract] OR

"aging"[Title/Abstract] OR

"older people"[Title/Abstract] OR

"older adult*"[Title/Abstract] OR

"older patient*"[Title/Abstract] OR

"old people"[Title/Abstract] OR

"old adult*"[Title/Abstract] OR

"old patient*"[Title/Abstract] OR

"old population"[Title/Abstract] OR

"old women"[Title/Abstract] OR

"old men"[Title/Abstract] OR

"older women"[Title/Abstract] OR

"older men"[Title/Abstract] OR

"older population"[Title/Abstract] OR

"old person*"[Title/Abstract] OR

"older person*"[Title/Abstract] OR

senior*[Title/Abstract] OR

"sarcopeni*"[Title/Abstract] OR

"Frail*"[Title/Abstract]

**Community-dwelling**

"Independent Living"[Mesh] OR

"Community health services"[Mesh] OR

"Primary health care"[Mesh] OR

aging in place[Title/Abstract] OR

Community-based[Title/Abstract] OR

Communitybased[Title/Abstract] OR

Community center*[Title/Abstract] OR

Community health services[Title/Abstract] OR

Community living[Title/Abstract] OR

Community population[Title/Abstract] OR

Community setting*[Title/Abstract] OR

community-dwell*[Title/Abstract] OR

community dwell*[Title/Abstract] OR

community-living*[Title/Abstract] OR

day center*[Title/Abstract] OR

home-based*[Title/Abstract] OR

home-dwell*[Title/Abstract] OR

home vist*[Title/Abstract] OR

Independent*[Title/Abstract] OR

Living in the community[Title/Abstract] OR

Outpatient*[Title/Abstract] OR

Participant home*[Title/Abstract] OR

Patient home*[Title/Abstract] OR

Primary health care[Title/Abstract]

**Physical performance outcome measures according to EWGSOP2**

"walking speed"[MeSH Terms] OR

"walking speed*"[Title/Abstract] OR

"walking pace*"[Title/Abstract] OR

((walk*[Title/Abstract] OR gait[Title/Abstract]) AND (test[Title/Abstract] OR speed[Title/Abstract] OR timed[Title/Abstract])) OR

"gait speed*"[Title/Abstract] OR

"gait analysis"[Title/Abstract] OR

"walk test"[Title/Abstract] OR

"walking test"[Title/Abstract] OR

"Timed up and go"[Title/Abstract] OR

"TUG"[Title/Abstract] OR

"SPPB"[Title/Abstract] OR

"short physical performance battery"[Title/Abstract] OR

"National Institute on Aging Battery"[Title/Abstract] OR

"NIA Battery"[Title/Abstract] OR

"Quartile Summary Physical Performance Score"[Title/Abstract]

**Psychometric properties (updated and adapted filter [1])**

"Validation Study"[Publication Type]

OR "reproducibility of results"[MeSH Terms]

OR "reproducib*"[Title/Abstract]

OR "psychometrics"[MeSH Terms]

OR "psychometr*"[Title/Abstract]

OR "clinimetr*"[Title/Abstract]

OR "clinometr*"[Title/Abstract]

OR "observer variation"[MeSH Terms]

OR "observer variation"[Title/Abstract]

OR "discriminant analysis"[MeSH Terms]

OR "reliab*"[Title/Abstract]

OR "valid*"[Title/Abstract]

OR "coefficient"[Title/Abstract]

OR "internal consistency"[Title/Abstract]

OR ("cronbach*"[Title/Abstract] AND ("alpha"[Title/Abstract] OR "alphas"[Title/Abstract]))

OR "item correlation"[Title/Abstract]

OR "item correlations"[Title/Abstract]

OR "item selection"[Title/Abstract]

OR "item selections"[Title/Abstract]

OR "item reduction"[Title/Abstract]

OR "item reductions"[Title/Abstract]

OR "agreement"[Text Word]

OR "precision"[Text Word]

OR "imprecision"[Text Word]

OR "precise values"[Text Word]

OR "test-retest"[Title/Abstract]

OR ("test"[Title/Abstract] AND "retest"[Title/Abstract])

OR ("reliab*"[Title/Abstract] AND ("test"[Title/Abstract] OR "retest"[Title/Abstract]))

OR "stability"[Title/Abstract]

OR "interrater"[Title/Abstract]

OR "inter-rater"[Title/Abstract]

OR "intrarater"[Title/Abstract]

OR "intra-rater"[Title/Abstract]

OR "intertester"[Title/Abstract]

OR "inter-tester"[Title/Abstract]

OR "intratester"[Title/Abstract]

OR "intra-tester"[Title/Abstract]

OR "interobserver"[Title/Abstract]

OR "inter-observer"[Title/Abstract]

OR "intraobserver"[Title/Abstract]

OR "intra-observer"[Title/Abstract]

OR "intertechnician"[Title/Abstract]

OR "inter-technician"[Title/Abstract]

OR "intratechnician"[Title/Abstract]

OR "intra-technician"[Title/Abstract]

OR "interexaminer"[Title/Abstract]

OR "inter-examiner"[Title/Abstract]

OR "intraexaminer"[Title/Abstract]

OR "intra-examiner"[Title/Abstract]

OR "interassay"[Title/Abstract]

OR "inter-assay"[Title/Abstract]

OR "intraassay"[Title/Abstract]

OR "intra-assay"[Title/Abstract]

OR "interindividual"[Title/Abstract]

OR "inter-individual"[Title/Abstract]

OR "intraindividual"[Title/Abstract]

OR "intra-individual"[Title/Abstract]

OR "interparticipant"[Title/Abstract]

OR "inter-participant"[Title/Abstract]

OR "intraparticipant"[Title/Abstract]

OR "intra-participant"[Title/Abstract]

OR "kappa"[Title/Abstract]

OR "kappa's"[Title/Abstract]

OR "kappas"[Title/Abstract]

OR "coefficient of variation"[Title/Abstract]

OR "repeatab*"[Text Word]

OR (("replicab*"[Text Word] OR "repeated"[Text Word]) AND ("measure"[Text Word] OR "measures"[Text Word] OR "findings"[Text Word] OR "result"[Text Word] OR "results"[Text Word] OR "test"[Text Word] OR "tests"[Text Word]))

OR "generaliza*"[Title/Abstract]

OR "generalisa*"[Title/Abstract]

OR "concordance"[Title/Abstract]

OR ("intraclass"[Title/Abstract] AND "correlation*"[Title/Abstract])

OR "discriminative"[Title/Abstract]

OR "known group"[Title/Abstract]

OR "factor analysis"[Title/Abstract]

OR "factor analyses"[Title/Abstract]

OR "factor structure"[Title/Abstract]

OR "factor structures"[Title/Abstract]

OR "dimensionality"[Title/Abstract]

OR "subscale*"[Title/Abstract]

OR "multitrait scaling analysis"[Title/Abstract]

OR "multitrait scaling analyses"[Title/Abstract]

OR "item discriminant"[Title/Abstract]

OR "interscale correlation"[Title/Abstract]

OR "interscale correlations"[Title/Abstract]

OR (("error"[Title/Abstract] OR "errors"[Title/Abstract]) AND ("measure*"[Title/Abstract] OR "correlat*"[Title/Abstract] OR "evaluat*"[Title/Abstract] OR "accuracy"[Title/Abstract] OR "accurate"[Title/Abstract] OR "precision"[Title/Abstract] OR "mean"[Title/Abstract]))

OR "accuracy"[Title/Abstract]

OR "accurate"[Title/Abstract]

OR "individual variability"[Title/Abstract]

OR "interval variability"[Title/Abstract]

OR "rate variability"[Title/Abstract]

OR "variability analysis"[Title/Abstract]

OR ("uncertainty"[Title/Abstract] AND ("measurement"[Title/Abstract] OR "measuring"[Title/Abstract]))

OR "standard error of measurement"[Title/Abstract]

OR "sensitiv*"[Title/Abstract]

OR "responsive*"[Title/Abstract]

OR ("limit"[Title/Abstract] AND "detection"[Title/Abstract])

OR "minimal detectable concentration"[Title/Abstract]

OR "interpretab*"[Title/Abstract]

OR ("small*"[Title/Abstract] AND ("real"[Title/Abstract] OR "detectable"[Title/Abstract]) AND ("change"[Title/Abstract] OR "difference"[Title/Abstract]))

OR "meaningful change"[Title/Abstract]

OR "minimal important change"[Title/Abstract]

OR "minimal important difference"[Title/Abstract]

OR "minimally important change"[Title/Abstract]

OR "minimally important difference"[Title/Abstract]

OR "minimal detectable change"[Title/Abstract]

OR "minimal detectable difference"[Title/Abstract]

OR "minimally detectable change"[Title/Abstract]

OR "minimally detectable difference"[Title/Abstract]

OR "minimal real difference"[Title/Abstract]

OR "ceiling effect"[Title/Abstract]

OR "floor effect"[Title/Abstract]

OR "Item response model"[Title/Abstract]

OR "IRT"[Title/Abstract]

OR "Rasch"[Title/Abstract]

OR "Differential item functioning"[Title/Abstract]

OR "DIF"[Title/Abstract]

OR "cross-cultural equivalence"[Title/Abstract]

**Exclusion filter [1]**

(‘‘address’’[Publication Type]

OR ‘‘biography’’[Publication Type]

OR ‘‘case reports’’[Publication Type]

OR ‘‘comment’’[Publication Type]

OR ‘‘directory’’[Publication Type]

OR ‘‘editorial’’[Publication Type]

OR ‘‘festschrift’’[Publication Type]

OR ‘‘interview’’[Publication Type]

OR ‘‘lecture’’[Publication Type]

OR ‘‘legal case’’[Publication Type]

OR ‘‘legislation’’[Publication Type]

OR ‘‘letter’’[Publication Type]

OR ‘‘news’’[Publication Type]

OR ‘‘newspaper article’’[Publication Type]

OR ‘‘patient education handout’’[Publication Type]

OR ‘‘popular work’’[Publication Type]

OR ‘‘congress’’ [Publication Type]

OR ‘‘consensus development conference’’[Publication Type]

OR ‘‘consensus development conference, nih’’[Publication Type]

OR ‘‘practice guideline’’[Publication Type]) NOT ("animals"[MeSH Terms] NOT "humans"[MeSH Terms])

**EMBASE**

**Older people**

Exp aged/ OR

exp aging/ OR

exp geriatrics/ OR

frail elderly/ OR

sarcopenia/ OR

"aged".ti,ab,kf OR

"ageing".ti,ab,kf OR

"aging".ti,ab,kf OR

"elder*".ti,ab,kf OR

"frail*".ti,ab,kf OR

"geriatric*".ti,ab,kf OR

"old adult*".ti,ab,kf OR

"old patient*".ti,ab,kf OR

"old people".ti,ab,kf OR

"old person*".ti,ab,kf OR

"old population".ti,ab,kf OR

"older adult*".ti,ab,kf OR

"older patient*".ti,ab,kf OR

"older people".ti,ab,kf OR

"older person*".ti,ab,kf OR

"older population".ti,ab,kf OR

"sarcopeni*".ti,ab,kf OR

"senior*".ti,ab,kf OR

"old men".ti,ab,kf OR

"old women".ti,ab,kf OR

"older men".ti,ab,kf OR

"older women".ti,ab,kf

**Community-dwelling**

community living/ OR

exp community care/ OR

exp community dwelling person/ OR

exp independent living/ OR

exp primary health care/ OR

"Community center*".ti,ab,kf OR

"Community health services".ti,ab,kf OR

"Community living".ti,ab,kf OR

"Community population".ti,ab,kf OR

"Community setting*".ti,ab,kf OR

"Communitybased".ti,ab,kf OR

"Community-based".ti,ab,kf OR

"community-dwell*".ti,ab,kf OR

"community-living*".ti,ab,kf OR "aging in place".ti,ab,kf OR

"day center*".ti,ab,kf OR

"home vist*".ti,ab,kf OR

"home-based*".ti,ab,kf OR

"home-dwell*".ti,ab,kf OR "Independent*".ti,ab,kf OR

"Living in the community".ti,ab,kf OR

"Outpatient*".ti,ab,kf OR

"Participant home*".ti,ab,kf OR

"Patient home*".ti,ab,kf OR

"Primary health care".ti,ab,kf

**Physical performance outcome measures according to EWGSOP2**

exp walking speed/ OR

"gait analysis".ti,ab,kf OR

"gait speed*".ti,ab,kf OR

"National Institute on Aging Battery".ti,ab,kf OR

"NIA Battery".ti,ab,kf OR

"Quartile Summary Physical Performance Score".ti,ab,kf OR

"short physical performance battery".ti,ab,kf OR

"SPPB".ti,ab,kf OR

"Timed up and go".ti,ab,kf OR

"TUG".ti,ab,kf OR

"walk test".ti,ab,kf OR

"walking pace*".ti,ab,kf OR

"walking speed*".ti,ab,kf OR

"walking test".ti,ab,kf OR

((walk*.ti,ab,kf OR gait.ti,ab,kf) AND (test.ti,ab,kf OR speed.ti,ab,kf OR timed.ti,ab,kf))

**Psychometric properties (updated, adapted and translated filter [1])**

exp intermethod comparison/

OR exp validation study/

OR exp psychometry/

OR exp reproducibility/

OR reproducib*.ti,ab,kf.

OR psychometr*.ti,ab,kf.

OR clinimetr*.ti,ab,kf.

OR clinometr*.ti,ab,kf.

OR exp observer variation/

OR observer variation.ti,ab,kf.

OR exp discriminant analysis/

OR exp validity/

OR reliab*.ti,ab,kf.

OR valid*.ti,ab,kf.

OR coefficient.ti,ab,kf.

OR internal consistency.ti,ab,kf.

OR (cronbach*.ti,ab,kf. AND (alpha.ti,ab,kf. OR alphas.ti,ab,kf.))

OR item correlation.ti,ab,kf.

OR item correlations.ti,ab,kf.

OR item selection.ti,ab,kf.

OR item selections.ti,ab,kf.

OR item reduction.ti,ab,kf.

OR item reductions.ti,ab,kf.

OR agreement.ti,ab,kf.

OR precision.ti,ab,kf.

OR imprecision.ti,ab,kf.

OR precise values.ti,ab,kf.

OR test-retest.ti,ab,kf.

OR (test.ti,ab,kf. AND retest.ti,ab,kf.)

OR (reliab*.ti,ab,kf. AND (test.ti,ab,kf. OR retest.ti,ab,kf.))

OR stability.ti,ab,kf.

OR interrater.ti,ab,kf.

OR inter-rater.ti,ab,kf.

OR intrarater.ti,ab,kf.

OR intra-rater.ti,ab,kf.

OR intertester.ti,ab,kf.

OR inter-tester.ti,ab,kf.

OR intratester.ti,ab,kf.

OR interobserver.ti,ab,kf.

OR inter-observer.ti,ab,kf.

OR intraobserver.ti,ab,kf.

OR intra-observer.ti,ab,kf.

OR intertechnician.ti,ab,kf.

OR inter-technician.ti,ab,kf.

OR intratechnician.ti,ab,kf.

OR intra-technician.ti,ab,kf.

OR interexaminer.ti,ab,kf.

OR inter-examiner.ti,ab,kf.

OR intraexaminer.ti,ab,kf.

OR intra-examiner.ti,ab,kf.

OR interassay.ti,ab,kf.

OR inter-assay.ti,ab,kf.

OR intraassay.ti,ab,kf.

OR intra-assay.ti,ab,kf.

OR interindividual.ti,ab,kf.

OR inter-individual.ti,ab,kf.

OR intraindividual.ti,ab,kf.

OR intra-individual.ti,ab,kf.

OR interparticipant.ti,ab,kf.

OR inter-participant.ti,ab,kf.

OR intraparticipant.ti,ab,kf.

OR intra-participant.ti,ab,kf.

OR kappa.ti,ab,kf.

OR kappas.ti,ab,kf.

OR coefficient of variation.ti,ab,kf.

OR repeatab*.ti,ab,kf.

OR (replicab*.ti,ab,kf. OR repeated.ti,ab,kf. AND (measure.ti,ab,kf. OR measures.ti,ab,kf. OR findings.ti,ab,kf. OR result.ti,ab,kf. OR results.ti,ab,kf. OR test.ti,ab,kf. OR tests.ti,ab,kf.))

OR generaliza*.ti,ab,kf.

OR generalisa*.ti,ab,kf.

OR concordance.ti,ab,kf.

OR (intraclass.ti,ab,kf. AND correlation*.ti,ab,kf.)

OR discriminative.ti,ab,kf.

OR known group.ti,ab,kf.

OR factor analysis.ti,ab,kf.

OR factor analyses.ti,ab,kf.

OR factor structure.ti,ab,kf.

OR factor structures.ti,ab,kf.

OR dimensionality.ti,ab,kf.

OR subscale*.ti,ab,kf.

OR multitrait scaling analysis.ti,ab,kf.

OR multitrait scaling analyses.ti,ab,kf.

OR item discriminant.ti,ab,kf.

OR interscale correlation.ti,ab,kf.

OR interscale correlations.ti,ab,kf.

OR (error.ti,ab,kf. OR errors.ti,ab,kf. AND (measure*.ti,ab,kf. OR correlat*.ti,ab,kf. OR evaluat*.ti,ab,kf. OR accuracy.ti,ab,kf. OR accurate.ti,ab,kf. OR precision.ti,ab,kf. OR mean.ti,ab,kf.))

OR accuracy.ti,ab,kf.

OR accurate.ti,ab,kf.

OR individual variability.ti,ab,kf.

OR interval variability.ti,ab,kf.

OR rate variability.ti,ab,kf.

OR variability analysis.ti,ab,kf.

OR (uncertainty.ti,ab,kf. AND (measurement.ti,ab,kf. OR measuring.ti,ab,kf.))

OR standard error of measurement.ti,ab,kf.

OR sensitiv*.ti,ab,kf.

OR responsive*.ti,ab,kf.

OR (limit.ti,ab,kf. AND detection.ti,ab,kf.)

OR minimal detectable concentration.ti,ab,kf.

OR interpretab*.ti,ab,kf.

OR (small*.ti,ab,kf. AND (real.ti,ab,kf. OR detectable.ti,ab,kf.) AND (change.ti,ab,kf. OR difference.ti,ab,kf.))

OR meaningful change.ti,ab,kf.

OR minimal important change.ti,ab,kf.

OR minimal important difference.ti,ab,kf.

OR minimally important change.ti,ab,kf.

OR minimally important difference.ti,ab,kf.

OR minimal detectable change.ti,ab,kf.

OR minimal detectable difference.ti,ab,kf.

OR minimally detectable change.ti,ab,kf.

OR minimally detectable difference.ti,ab,kf.

OR minimal real change.ti,ab,kf.

OR minimal real difference.ti,ab,kf.

OR minimally real change.ti,ab,kf.

OR minimally real difference.ti,ab,kf.

OR ceiling effect.ti,ab,kf.

OR floor effect.ti,ab,kf.

OR item response model.ti,ab,kf.

OR irt.ti,ab,kf.

OR rasch.ti,ab,kf.

OR differential item functioning.ti,ab,kf.

OR dif.ti,ab,kf.

OR computer adaptive testing.ti,ab,kf.

OR cross-cultural equivalence.ti,ab,kf.

**CINAHL**

**Older people**

MH "Aged+" OR

MH "Aging+" OR

MH "Frail Elderly" OR

MH "Geriatric Functional Assessment" OR

MH "Geriatrics+" OR

MH "Sarcopenia" OR

AB "aged" OR

AB "ageing" OR

AB "aging" OR

AB "elder*" OR

AB "frail*" OR

AB "geriatric*" OR

AB "old adult*" OR

AB "old patient*" OR

AB "old people" OR

AB "old person*" OR

AB "old population" OR

AB "older adult*" OR

AB "older patient*" OR

AB "older people" OR

AB "older person*" OR

AB "older population" OR

AB "sarcopeni*" OR

AB "senior*" OR

AB "old men" OR

AB "old women" OR

AB "older men" OR

AB "older women" OR

TI "aged" OR

TI "ageing" OR

TI "aging" OR

TI "elder*" OR

TI "frail*" OR

TI "geriatric*" OR

TI "old adult*" OR

TI "old patient*" OR

TI "old people" OR

TI "old person*" OR

TI "old population" OR

TI "older adult*" OR

TI "older patient*" OR

TI "older people" OR

TI "older person*" OR

TI "older population" OR

TI "sarcopeni*" OR

TI "senior*" OR

TI "old men" OR

TI "old women" OR

TI "older men" OR

TI "older women"

**Community-dwelling**

MH "Community Health Services" OR

MH "Community Living+" OR

MH "Primary Health Care" OR

AB aging in place OR

AB Community center* OR

AB Community health services OR

AB Community living OR

AB Community setting* OR

AB Communitybased OR

AB Community-based OR

AB community-dwell* OR

AB community-living* OR

AB day center* OR

AB home vist* OR

AB home-based* OR

AB home-dwell* OR

AB Independent* OR

AB Living in the community OR

AB Outpatient* OR

AB Participant home* OR

AB Patient home* OR

AB Primary health care OR

TI aging in place OR

TI Community center* OR

TI Community health services OR

TI Community living OR

TI community living OR
AB community living

TI Community setting* OR

TI Communitybased OR

TI Community-based OR

TI community-dwell* OR

TI community-living*

TI day center* OR

TI home vist* OR

TI home-based* OR

TI home-dwell* OR

TI Independent* OR

TI Living in the community OR

TI Outpatient* OR

TI Participant home* OR

TI Patient home* OR

TI Primary health care

**Physical performance outcome measures according to EWGSOP2**

MH "Walking Speed" OR

((AB walk* OR AB gait OR TI walk* OR TI gait) AND (AB test OR AB speed OR AB timed OR TI test OR TI speed OR TI timed)) OR

AB "gait analysis" OR

AB "gait speed*" OR

AB "National Institute on Aging Battery"[Title/Abstract] OR

AB "NIA Battery" OR

AB "Quartile Summary Physical Performance Score" OR

AB "short physical performance battery" OR

AB "SPPB" OR

AB "timed up and go" OR

AB "TUG" OR

AB "walk test" OR

AB "walking pace*" OR

AB "walking speed*" OR

AB "walking test" OR

TI "gait analysis" OR

TI "gait speed*" OR

TI "National Institute on Aging Battery"[Title/Abstract] OR

TI "NIA Battery" OR

TI "Quartile Summary Physical Performance Score" OR

TI "short physical performance battery" OR

TI "SPPB" OR

TI "Timed up and go" OR

TI "TUG" OR

TI "walk test" OR

TI "walking pace*" OR

TI "walking speed*" OR

TI "walking test"

**Psychometric properties (updated, adapted and translated filter [1])**

(MH "Validation Studies")

OR (MH "Psychometrics")

OR (MH "Reproducibility of Results")

OR (MH "Discriminant Analysis")

OR (MH "Internal Consistency+")

OR (MH "Reliability+")

OR (MH "Measurement Error+")

OR (MH "Reliability and Validity+")

OR (MH "Observer Bias+")

OR TI psychometr*

OR TI observer variation

OR TI Clinimetr*

OR TI Clinometr*

OR TI reproducib*

OR TI reliab*

OR TI un.r.eliab*

OR TI valid*

OR TI coefficient

OR TI "internal consistency"

OR AB psychometr*

OR AB Clinimetr*

OR AB Clinometr*

OR AB observer variation

OR AB reproducib*

OR AB reliab*

OR AB un.r.eliab*

OR AB valid*

OR AB coefficient

OR AB "internal consistency"

OR (TI cronbach* OR AB cronbach* AND (TI alpha OR AB alpha OR TI alphas OR AB alphas))

OR (TI item OR AB item AND (TI correlation* OR AB correlation* OR TI selection* OR AB selection* OR TI reduction* OR AB reduction*))

OR TI agreement

OR TI precision

OR TI imprecision

OR TI "precise values"

OR TI test-retest

OR AB agreement

OR AB precision

OR AB imprecision

OR AB "precise values"

OR AB test-retest

OR (TI test OR AB test AND (TI retest OR AB retest))

OR (TI reliab* OR AB reliab* AND (TI test OR AB test OR TI retest or AB retest))

OR TI stability

OR TI interrater

OR TI inter-rater

OR TI intrarater

OR TI intra-rater

OR TI intertester

OR TI inter-tester

OR TI intratester

OR TI intra-tester

OR TI interobserver

OR TI inter-observer

OR TI intraobserver

OR TI intra-observer

OR TI intertechnician

OR TI inter-technician

OR TI intratechnician

OR TI intra-technician

OR TI interexaminer

OR TI inter-examiner

OR TI intraexaminer

OR TI intra-examiner

OR TI interassay

OR TI inter-assay

OR TI intraassay

OR TI intra-assay

OR TI interindividual

OR TI inter-individual

OR TI intraindividual

OR TI intra-individual

OR TI interparticipant

OR TI inter-participant

OR TI intraparticipant

OR TI intra-participant

OR TI kappa

OR TI kappa’s

OR TI kappas

OR TI repeatab*

OR AB stability

OR AB interrater

OR AB inter-rater

OR AB intrarater

OR AB intra-rater

OR AB intertester

OR AB inter-tester

OR AB intratester

OR AB intra-tester

OR AB interobserver

OR AB inter-observer

OR AB intraobserver

OR AB intra-observer

OR AB intertechnician

OR AB inter-technician

OR AB intratechnician

OR AB intra-technician

OR AB interexaminer

OR AB inter-examiner

OR AB intraexaminer

OR AB intra-examiner

OR AB interassay

OR AB inter-assay

OR AB intraassay

OR AB intra-assay

OR AB interindividual

OR AB inter-individual

OR AB intraindividual

OR AB intra-individual

OR AB interparticipant

OR AB inter-participant

OR AB intraparticipant

OR AB intra-participant

OR AB kappa

OR AB kappa’s

OR AB kappas

OR AB repeatab*

OR ((TI replicab* OR AB replicab* OR TI repeated OR AB repeated) AND (TI measure OR AB measure OR TI measures OR AB measures OR TI findings OR AB findings OR TI result OR AB result OR TI results OR AB results OR TI test OR AB test OR TI tests OR AB tests))

OR TI generaliza*

OR TI generalisa*

OR TI concordance

OR AB generaliza*

OR AB generalisa*

OR AB concordance

OR (TI intraclass OR AB intraclass) AND (TI correlation* or AB correlation*))

OR TI discriminative

OR TI "known group"

OR TI factor analysis

OR TI factor analyses

OR TI dimensionality

OR TI subscale*

OR AB discriminative

OR AB "known group"

OR AB factor analysis

OR AB factor analyses

OR AB dimensionality

OR AB subscale*

OR ((TI multitrait OR AB multitrait) AND (TI scaling OR AB scaling) AND (TI analysis OR AB analysis OR TI analyses OR AB analyses))

OR TI item discriminant

OR TI interscale correlation*

OR TI "individual variability"

OR AB item discriminant

OR AB interscale correlation*

OR AB "individual variability"

OR (TI variability OR AB variability AND) (TI analysis OR AB analysis OR TI values OR AB values OR TI interval OR AB interval OR TI rate OR AB rate))

OR (TI uncertainty OR AB uncertainty) AND (TI measurement OR AB measurement OR TI measuring OR AB measuring))

OR TI "standard error of measurement"

OR TI sensitiv*

OR TI responsive*

OR AB "standard error of measurement"

OR AB sensitiv*

OR AB responsive*

OR (AB error OR AB errors AND (AB measure* OR AB correlat* OR AB evaluat* OR AB accuracy OR AB accurate OR AB precision OR AB mean.))

OR (TI error OR TI errors AND (TI measure* OR TI correlat* OR TI evaluat* OR TI accuracy OR TI accurate OR TI precision OR TI mean.))

OR AB accuracy

OR AB accurate

OR TI accuracy

OR TI accurate

OR ((TI minimal OR TI minimally OR TI clinical OR TI clinically OR AB minimal OR AB minimally OR AB clinical OR AB clinically) AND (TI important OR TI significant OR TI detectable OR AB important OR AB significant OR AB detectable) AND (TI change OR AB change OR TI difference OR AB difference OR TI concentration OR AB concentration))

OR (TI small* OR AB small* AND (TI real OR AB real OR TI detectable OR AB detectable) AND (TI change OR AB change OR TI difference OR AB difference))

OR TI meaningful change

OR TI "ceiling effect"

OR TI "floor effect"

OR TI "Item response model"

OR TI IRT

OR TI Rasch

OR TI "Differential item functioning"

OR TI DIF

OR TI "computer adaptive testing"

OR TI "cross-cultural equivalence"

OR AB meaningful change

OR AB "ceiling effect"

OR AB "floor effect"

OR AB "Item response model"

OR AB IRT

OR AB Rasch

OR AB "Differential item functioning"

OR AB DIF

OR AB "computer adaptive testing"

OR AB "cross-cultural equivalence"

OR TI "factor structure"

OR TI "factor structures"

OR AB "factor structure"

OR AB "factor structures"

OR ((TI limit OR AB limit) AND (TI detection OR AB detection))

OR TI interpretab*

OR AB interpretab*

**Appendix B: Description of instruments**

### **Short physical performance battery**

The SPPB is a compound test aimed to measure physical performance. It comprises of a balance test, a walk test and 5-times chair rise test [2]. In the original SPPB designed in 1994 participants walk 8 feet. However, the test has since been adapted and can also be performed using a 3- and 4-meter walk test [3]. The minimum SPPB score indicating mobility disability is 0, the maximum score indicating no disability is 12.

### **Timed up and go test**

During the TUG a participant begins seated, and is timed to stand up, walk 3 meters, turn around, walk back and sit back down [4, 5]. While standing up, participants should refrain from using their arms. Therefore, this test mostly relies on the lower limbs. The less time a person takes to complete the TUG, the better their physical performance.

### **4m gait speed test**

The 4m GST is used to evaluate lower extremity functioning. The participant is timed completing a 4-meter walk at their usual pace. The less time a person needs to walk the 4m GST, the better their physical performance.

### **400m walk test**

The 400m WT assesses a participant's walking ability and endurance. The participant is asked to complete 20 laps of 20m each, as fast as possible. They are allowed up to two rest stops during this test. The less time a person needs to perform the 400m WT, the better their physical performance.

**Appendix C: Overall rating methodology**

Test-retest, inter, or intra-reliability was deemed sufficient if the intra-class correlation coefficient (ICC) ≥ 0.70 [6]. Measurement error was rated sufficient when the minimal detectable change (MDC) < minimally (clinical) important difference (M(C)IC) [6]. Criterion validity was deemed sufficient when the area under the curve (AUC) ≥ 0.70 [7]. Construct validity analysis was performed for each measurement instrument. When multiple constructs were evaluated, groups were made of similar constructs and methods used for synthesis. Construct validity measured by ICC, Spearman's rank correlation coefficient (SRCC), Pearson correlation coefficient (PCC) was rated on sufficiency using hypothesis testing. Hypotheses were formed for comparators in the following categories: measuring the same construct (correlation coefficient >0.7), measuring a related construct (correlation coefficient 0.4-0.7), or measuring a different construct (correlation coefficient <0.4). The construct validity of an instrument was rated as sufficient when 75% of the hypotheses were accurate for a physical performance measure and comparators [7]. Lastly, responsiveness was deemed sufficient when at least 75% of studies showed a standard error of measurement (SEM)<meaningful change or AUC ≥ 0.70 [7].

**Appendix D: Mean values of physical performance tests**

*Table 7 SPPB mean values and standard deviations*

| First author | Subgroup | Mean score | SD |
| --- | --- | --- | --- |
| Balachandran [8] | Total | 10.60 | 2.10 |
| Balasubramanian [9] | Total | 10.50 | 1.60 |
| Bean [10] | Total | 8.67 | 1.50 |
| Fusco [11] | Total | 8.06 | 2.84 |
| Gómez [12] | Total | 9.70 | 2.00 |
| Gray [13] | High functioning | 11.49 | 0.71 |
|  | Low functioning | 7.88 | 1.09 |
|  | Men | 11.37 | 0.96 |
|  | Women | 10.12 | 1.98 |
| Lee [14] | Sarcopenia AWGS19 GS | 11.40 | 1.50 |
|  | Sarcopenia AWGS19 SPPB | 11.10 | 1.50 |
|  | Sarcopenia AWGS19 STS | 11.00 | 1.60 |
|  | Severe sarcopenia AWGS19 GS | 10.30 | 1.90 |
|  | Severe sarcopenia AWGS19 SPPB | 8.00 | 1.70 |
|  | Severe sarcopenia AWGS19 STS | 9.00 | 2.00 |
| Löppönen [15] | Men 75-80 y/o | 10.80 | 1.60 |
|  | Men 80-85 y/o | 10.50 | 2.00 |
|  | Men 85+ y/o | 9.70 | 1.90 |
|  | Women 75-80 y/o | 10.50 | 1.60 |
|  | Women 80-85 y/o | 10.40 | 1.80 |
|  | Women 85+ y/o | 9.20 | 2.20 |
| Mathis [16] | Test 1 | 8.10 | 2.70 |
|  | Test 2 | 8.50 | 2.60 |
| Ni [17] | Total | 8.60 | 1.80 |
| Perera [18] | Total | 9.30 | 2.70 |
| Portegijs [19] | Light self-reported physical activity | 9.00 | 5.00 |
|  | Moderate self-reported physical activity | 11.00 | 2.00 |
|  | Regular self-reported physical activity | 11.00 | 2.00 |
| Riwniak [20] | Total | 10.70 | 2.10 |
| Stanziano [21] | Total | n.r. | n.r. |

*Table 8 TUG mean values and standard deviations*

| First author | Subgroup | Mean time (s) | SD |
| --- | --- | --- | --- |
| Alcock [22] | Total | 7.85 | 2.90 |
| Balachandran [8] | Fast | 6.60 | 2.50 |
|  | Normal | 8.50 | 2.30 |
| Balasubramanian [9] | Total | 10.50 | 2.20 |
| Beauchamp [23] | Test 1 | 10.47 | 2.17 |
|  | Test 2 | 10.07 | 2.20 |
| Cho [24] | Total | 15.00 | 8.00 |
| Creel [25] | Total | n.r. | n.r. |
| de Vreede [26] | Total | 6.00 | 1.90 |
| Di Fabio [27] | Total | n.r. | n.r. |
| Gamerman [28] | Total | n.r. | n.r. |
| Goldberg [29] | Total | 12.30 | 0.50 |
| Gordt [30] | Total | 11.50 | 5.10 |
| Griswold [31] | Total | n.r. | n.r. |
| Hachiya [32] | Total | 6.70 | 1.40 |
| Härdi [33] | Total | 10.90 | 2.50 |
| Hashidate [34] | Total | 24.10 | 13.10 |
| Kim [35] | No mobility limitations | 6.21 | 1.10 |
|  | Moderate mobility limitations | 8.35 | 2.60 |
|  | Severe mobility limitations | 10.50 | 4.60 |
| Kristensen [36] | Rater 1 | 10.80 | 4.80 |
|  | Rater 2 | 11.00 | 4.70 |
| Kwan [37] | Total | 10.90 | 3.68 |
| Lin [38] | Total | 13.30 | n.r. |
| Looijaard [39] | No mobility limitations | 6.21 | 1.10 |
|  | Moderate mobility limitations | 8.35 | 2.60 |
|  | Severe mobility limitations | 10.50 | 4.60 |
| Mathis [16] | Test 1 | 11.40 | 4.70 |
|  | Test 2 | 10.90 | 4.60 |
| Minematsu [40] | Men | 6.65 | 1.22 |
|  | Women | 6.96 | 1.38 |
| Nepal [41] | Total | 14.80 | 6.00 |
| O'Hoski [42] | Total | 8.70 | 1.90 |
| Olivares [43] | Total | n.r. | n.r. |
| Özden [44] | Total | 7.94 | 2.27 |
| Schaubert [45] | Total | n.r. | n.r. |
| Schepens [46] | Total | 12.35 | 0.49 |
| Steffen [47] | Men 60-69 y/o | 8.00 | 2.00 |
|  | Men 70-79 y/o | 9.00 | 3.00 |
|  | Men 80-89 y/o | 10.00 | 1.00 |
|  | Women 60-69 y/o | 8.00 | 2.00 |
|  | Women 70-79 y/o | 9.00 | 2.00 |
|  | Women 80-89 y/o | 11.00 | 3.00 |
| Suwannarat [48] | Caregivers | 12.01 | 2.98 |
|  | Health volunteers | 13.06 | 3.48 |
|  | Physical therapists | 12.32 | 3.39 |
| Suzuki [49] | Test 1 | 6.00 | 1.00 |
|  | Test 2 | 5.80 | 0.90 |
| Wang [50] | Total | 8.60 | 1.80 |
| Wrisley [51] | Total | 10.90 | 4.10 |

*Table 9 4m GST mean values and standard deviations*

| First author | Subgroup | Mean speed (m/s) | SD |
| --- | --- | --- | --- |
| Bean [10] | Total | 0.93 | 0.23 |
| Beauchamp [23] | Test 1 | 0.90 | 0.16 |
|  | Test 2 | 0.91 | 0.15 |
| Fernández-Huerta [52] | Test 1 | 1.57 | 0.33 |
|  | Test 2 | 1.57 | 0.35 |
| Fusco [11] | Total | 0.85 | 0.30 |
| Goldberg [53] | Total | 1.08 | 0.27 |
|  | Intermediate gait speed | 0.85 | 0.10 |
|  | Fast gait speed | 1.30 | 0.19 |
| Maggio [54] | Total | n.r. | n.r. |
| Pasma [55] | Total | 0.72 | 0.27 |
| Perera [18] | Total | 0.88 | 0.24 |
| Riwniak [20] | Total | 1.00 | 0.20 |
| Rolland [56] | Total | 0.77 | 0.24 |
| van Ancum [57] | Total | 1.43 | 0.21 |

*Table 10 Median (IQR) values 400m WT*

| First author | Subgroup | Median | IQR |
| --- | --- | --- | --- |
| Rolland [56] | Total | n.r. | n.r. |
| Simonsick [58] | Men (speed: m/s) | 1.30 | 1.15-1.43 |
|  | Women (speed: m/s) | 1.19 | 1.06-1.31 |
|  | Men (time: min) | 5.09 | 4.39-5.48 |
|  | Women (time: min) | 5.36 | 5.06-6.16 |

**Appendix E: Risk of Bias Assessment**

*Table 11 Risk of Bias Assessment of each study. COSMIN Risk of Bias tool [59] used for reliability and measurement error. COSMIN Risk of Bias checklist [60] used for validity and responsiveness.*

| Reference | Reliability | Measurement error | Criterion validity | Hypothesis testing for construct validity | Responsiveness |
| --- | --- | --- | --- | --- | --- |
| Alcock [22] | N/A | N/A | N/A | Adequate | N/A |
| Balachandran [8] | N/A | N/A | N/A | Inadequate | N/A |
| Balasubramanian [9] | N/A | N/A | N/A | Very good | N/A |
| Bean [10] | N/A | N/A | N/A | Very good | N/A |
| Beauchamp [23] | Adequate | Adequate | N/A | N/A | N/A |
| Cho [24] | N/A | N/A | N/A | Inadequate | N/A |
| Creel [25] | Doubtful | N/A | N/A | Very good | N/A |
| de Vreede [26] | N/A | N/A | N/A | Very good | N/A |
| Di Fabio [27] | N/A | N/A | N/A | Very good | N/A |
| Fernández-Huerta [52] | Inadequate | N/A | N/A | Inadequate | N/A |
| Fusco [11] | N/A | N/A | N/A | Inadequate | N/A |
| Gamerman [28] | N/A | N/A | N/A | Inadequate | N/A |
| Goldberg [53] | Doubtful | Doubtful | N/A | N/A | N/A |
| Goldberg [29] | N/A | N/A | N/A | Very good | N/A |
| Gómez [12] | Inadequate | N/A | N/A | N/A | N/A |
| Gordt [30] | N/A | N/A | N/A | Very good | N/A |
| Gray [13] | N/A | N/A | N/A | Doubtful | N/A |
| Griswold [31] | Doubtful | Doubtful | N/A | Doubtful | N/A |
| Hachiya [32] | N/A | N/A | N/A | Very good | N/A |
| Härdi [33] | N/A | N/A | N/A | Doubtful | N/A |
| Hashidate [34] | N/A | N/A | N/A | Doubtful | N/A |
| Kim [35] | N/A | N/A | Inadequate | N/A | N/A |
| Kristensen [36] | Very good | Very good | N/A | N/A | N/A |
| Kwan [37] | N/A | N/A | N/A | Doubtful | N/A |
| Lee [14] | N/A | N/A | Very good | N/A | N/A |
| Lin [38] | N/A | N/A | Inadequate | Inadequate | Inadequate |
| Looijaard [39] | N/A | N/A | N/A | Very good | N/A |
| Löppönen [15] | N/A | N/A | N/A | Doubtful | N/A |
| Maggio [54] | N/A | N/A | N/A | Doubtful | N/A |
| Mathis [16] | Adequate | Adequate | N/A | Adequate | N/A |
| Minematsu [40] | N/A | N/A | N/A | Inadequate | N/A |
| Nepal [41] | Very good | N/A | N/A | N/A | N/A |
| Ni [17] | N/A | N/A | N/A | Very good | N/A |
| O'Hoski [42] | N/A | N/A | N/A | Very good | N/A |
| Olivares [43] | N/A | N/A | N/A | Very good | N/A |
| Özden [44] | N/A | N/A | N/A | Very good | N/A |
| Pasma [55] | N/A | N/A | N/A | Inadequate | N/A |
| Perera [18] | N/A | N/A | N/A | N/A | Very good |
| Portegijs [15, 19] | N/A | N/A | N/A | Adequate | N/A |
| Riwniak [20] | N/A | N/A | N/A | Doubtful | N/A |
| Rolland [56] | Doubtful | N/A | N/A | Very good | N/A |
| Schaubert [45] | N/A | N/A | N/A | Very good | N/A |
| Schepens [46] | N/A | N/A | N/A | Very good | N/A |
| Simonsick [58] | N/A | N/A | N/A | Inadequate | N/A |
| Stanziano [21] | N/A | N/A | N/A | Inadequate | N/A |
| Steffen [47] | Doubtful | N/A | N/A | N/A | N/A |
| Suwannarat [48] | Doubtful | N/A | N/A | N/A | N/A |
| Suzuki [49] | Doubtful | Doubtful | N/A | N/A | N/A |
| Wang [50] | N/A | N/A | N/A | Very good | N/A |
| Wrisley [51] | N/A | N/A | N/A | Very good | N/A |

**Appendix F: GRADE Evaluation**

*Table 12 evaluation: Reliability*

|  | Risk of Bias | Inconsistency | Imprecision | Indirectness |
| --- | --- | --- | --- | --- |
| SPPB | Serious (-1) | No | No | No |
| TUG | No | No | No | No |
| 4M GST | Serious (-1) | No | No | No |
| 400M WT | N/A | N/A | N/A | N/A |

*Table 13 GRADE evaluation: Measurement error*

|  | Risk of Bias | Inconsistency | Imprecision | Indirectness |
| --- | --- | --- | --- | --- |
| SPPB | Serious (-1) | No | Very serious (-2) | No |
| TUG | No | No | No | No |
| 4M GST | Serious (-1) | No | No | No |
| 400M WT | N/A | N/A | N/A | N/A |

*Table 14 GRADE evaluation: Criterion validity*

|  | Risk of Bias | Inconsistency | Imprecision | Indirectness |
| --- | --- | --- | --- | --- |
| SPPB | No | No | No | Serious (-1) |
| TUG | Very serious (-2) | No | No | No |
| 4M GST | N/A | N/A | N/A | N/A |
| 400M WT | N/A | N/A | N/A | N/A |

*Table 15 GRADE evaluation: Construct validity*

|  | Risk of Bias | Inconsistency | Imprecision | Indirectness |
| --- | --- | --- | --- | --- |
| SPPB | No | Serious (-1) | No | No |
| TUG | No | No | No | No |
| 4M GST | No | Serious (-1) | No | No |
| 400M WT | No | Serious (-1) | No | No |

*Table 16 GRADE evaluation: Responsiveness*

| Responsiveness | Risk of Bias | Inconsistency | Imprecision | Indirectness |
| --- | --- | --- | --- | --- |
| SPPB | No | No | No | No |
| TUG | Very serious (-2) | No | No | Serious (-1) |
| 4M GST | No | No | No | No |
| 400M WT | N/A | N/A | N/A | N/A |

**Appendix G: Construct validity comparator groups**

Balance: 3m backward walk, activities-specific balance confidence, activity-specific balance confidence skill, Berg balance scale, BESTest (including briefBESTest and mini-BESTest), functional reach, maximal step length, maximal step length, modified total body rotation test, narrow walk score, rapid step test, standing balance score, tandem stance time, Tinetti balance, TUG (and 3f up-and-go), TURN180 and unipedal stance time.

Chair stand tests: chair stand test, sit to stand test.

Mobility: performance-oriented mobility assessment and timed movement battery.

Muscle strength: double leg press power, handgrip strength, knee extension strength, knee flexion strength, leg press power and stair climb power.

Physical functioning questionnaires: activities of daily living, assessment of daily activity performance, community balance and mobility scale (English and German), elderly physical function scale, fast evaluation of mobility, balance and fear, life-space assessment, life-space mobility at home, patient-specific functional scale, self-reported health, self-reported vitality and SF-36 physical component.

Sarcopenia: diagnostic validity sarcopenia (Baumgarter, EWGSOP, FNIH, IWGS, Janssen).

Short walking test: 3m, 3f, 4m, 6m, 10m, 20m, 50m, 50ft, 400m and 2 minute walking tests, functional gait assessment and Tinetti gait assessment.

Long walking test: 6 minute walking test.

Rest: Back Scratch, Bodily Pain, Fast Evaluation of Mobility, Balance, and Fear (fear complaints), Fast Evaluation of Mobility, Balance, and Fear (risk factors), Fear of Falling, Gariatric Depression Scale Score, MMSE, Number of Free Living Sit to Stand and Sit and Reach.

# Appendix H: Construct validity results

Table S 1 Construct validity of SPPB.

| First author | Comparator group | Comparator | Method | Value | P value | 95%CI lower | 95%CI upper | SD |
| --- | --- | --- | --- | --- | --- | --- | --- | --- |
| Gray [13] | Balance | 8f Up and Go | PCC | -0.700 | <0.05 | n.r. | n.r. | n.r. |
| Stanziano [21] | Balance | Modified Total Body Rotation Test | PCC | 0.307 | 0.002 | n.r. | n.r. | n.r. |
| Balachandran [8] | Chair stand tests | Sit to Stand | PCC | 0.410 | n.r. | 0.17 | 0.59 | n.r. |
| Gray [13] | Chair stand tests | Chair Stand Test | PCC | 0.690 | <0.05 | n.r. | n.r. | n.r. |
| Bean [10] | Muscle strength | Stair Climb Power | SRCC | 0.510 | <0.001 | n.r. | n.r. | n.r. |
| Ni [17] | Muscle strength | Stair Climb Power (10 Steps) | PCC | 0.220 | 0.039 | n.r. | n.r. | n.r. |
| Ni [17] | Muscle strength | Stair Climb Power (4 Steps) | PCC | 0.190 | 0.041 | n.r. | n.r. | n.r. |
| Balachandran [8] | Muscle strength | Leg Press Power | PCC | 0.290 | n.r. | 0.07 | 0.51 | n.r. |
| Bean [10] | Muscle strength | Double Leg Press Power (40% one rep maximum) | SRCC | 0.420 | <0.001 | n.r. | n.r. | n.r. |
| Bean [10] | Muscle strength | Double Leg Press Power (70% one rep maximum) | SRCC | 0.440 | <0.001 | n.r. | n.r. | n.r. |
| Balasubramanian [9] | Questionnaire | Community Balance and Mobility Scale | SRCC | 0.750 | <0.001 | n.r. | n.r. | n.r. |
| Fusco [11] | Questionnaire | Activities of Daily Living | SRCC | 0.478 | <0.001 | n.r. | n.r. | n.r. |
| Fusco [11] | Questionnaire | Instrumental Activities of Daily Living | SRCC | 0.626 | <0.001 | n.r. | n.r. | n.r. |
| Mathis [16] | Questionnaire | Patient Specific Functional Scale | PCC | 0.370 | 0.030 | n.r. | n.r. | n.r. |
| Portegijs [19] | Questionnaire | Self-Reported Scale Assessing Habitual Physical Activity | SRCC | 0.400 | <0.001 | n.r. | n.r. | n.r. |
| Riwniak [20] | Questionnaire | Neuro-QOL Lower Extremity-Mobility Function | PCC | 0.290 | <0.05 | n.r. | n.r. | n.r. |
| Fusco [11] | Short walking test | 4m Gait Speed | SRCC | 0.776 | <0.001 | n.r. | n.r. | n.r. |
| Gray [13] | Short walking test | 3f Walking Velocity (Maximal Speed) | PCC | 0.350 | <0.05 | n.r. | n.r. | n.r. |
| Gray [13] | Short walking test | 3f Walking Velocity (Usual Speed) | PCC | 0.190 | n.r. | n.r. | n.r. | n.r. |
| Gray [13] | Long walking test | 6 Minute Walk | PCC | 0.630 | <0.05 | n.r. | n.r. | n.r. |
| Löppönen [15] | Rest | Number of Free Living Sit to Stand | SRCC | 0.170 | <0.001 | n.r. | n.r. | n.r. |

Table S 2 Construct validity of TUG.

| First author | Comparator group | Comparator | Method | Value | P value | 95%CI lower | 95%CI upper | SD |
| --- | --- | --- | --- | --- | --- | --- | --- | --- |
| Gamerman [28] | Balance | TURN180 (Total) | SRCC | 0.881 | <0.001 | n.r. | n.r. | n.r. |
| Griswold [31] | Balance | TUG VR | PCC | 0.878 | <0.01 | n.r. | n.r. | n.r. |
| O'Hoski [42] | Balance | BESTest | PCC | 0.680 | <0.001 | n.r. | n.r. | n.r. |
| O'Hoski [42] | Balance | briefBESTest | PCC | -0.600 | <0.001 | n.r. | n.r. | n.r. |
| O'Hoski [42] | Balance | mini-BESTest | PCC | -0.066 | <0.001 | n.r. | n.r. | n.r. |
| Olivares [43] | Balance | Functional Reach | PCC | -0.386 | <0.001 | n.r. | n.r. | n.r. |
| Cho [24] | Balance | Activity-Specific Balance Confidence Skill | SRCC | -0.606 | <0.001 | n.r. | n.r. | n.r. |
| Cho [24] | Balance | Tandem Stance Time | SRCC | -0.485 | <0.001 | n.r. | n.r. | n.r. |
| Cho [24] | Balance | Tandem Stance Time | SRCC | 0.564 | <0.001 | n.r. | n.r. | n.r. |
| Cho [24] | Balance | Unipedal Stance Time | SRCC | -0.558 | <0.001 | n.r. | n.r. | n.r. |
| Lin [38] | Balance | Tinetti Balance | n.r. Convergent | -0.550 | n.r. | n.r. | n.r. | n.r. |
| Schepens [46] | Balance | Activities-Specific Balance Confidence (16) | PCC | -0.650 | <0.001 | n.r. | n.r. | n.r. |
| Schepens [46] | Balance | Activities-Specific Balance Confidence (6) | PCC | -0.690 | <0.001 | n.r. | n.r. | n.r. |
| Wang [50] | Balance | Berg Balance Scale | SRCC | -0.530 | <0.01 | n.r. | n.r. | n.r. |
| Goldberg [29] | Balance | Maximal Step Length | PCC | -0.650 | <0.01 | n.r. | n.r. | n.r. |
| Cho [24] | Balance | Maximal Step Length | SRCC | -0.679 | <0.001 | n.r. | n.r. | n.r. |
| Cho [24] | Balance | Rapid Step Test | SRCC | 0.346 | <0.001 | n.r. | n.r. | n.r. |
| Özden [44] | Balance | 3m Backward Walk (Test 1) | SRCC | 0.649 | <0.01 | n.r. | n.r. | n.r. |
| Özden [44] | Balance | 3m Backward Walk (Test 2) | SRCC | 0.645 | <0.01 | n.r. | n.r. | n.r. |
| Alcock [22] | Chair stand tests | Sit to Stand | PCC | 0.380 | <0.05 | n.r. | n.r. | n.r. |
| Balachandran [8] | Chair stand tests | Sit to Stand | PCC | 0.370 | n.r. | -0.57 | -0.05 | n.r. |
| Schaubert [45] | Chair stand tests | Sit to Stand (Baseline) | PCC | 0.734 | <0.05 | n.r. | n.r. | n.r. |
| Schaubert [45] | Chair stand tests | Sit to Stand (Week 12) | PCC | 0.918 | <0.01 | n.r. | n.r. | n.r. |
| Schaubert | Chair stand tests | Sit to Stand (Week 6) | PCC | 0.882 | <0.01 | n.r. | n.r. | n.r. |
| Cho [24] | Mobility | Performance-Oriented Mobility Assessment | SRCC | -0.651 | <0.001 | n.r. | n.r. | n.r. |
| Creel [25] | Mobility | Timed Movement Battery (Max-Movement Speed) | PCC | 0.790 | <0.001 | n.r. | n.r. | n.r. |
| Creel [25] | Mobility | Timed Movement Battery (Self-Selected Speed) | PCC | 0.890 | <0.001 | n.r. | n.r. | n.r. |
| Balachandran [8] | Muscle strength | Leg Press Power | PCC | -0.290 | n.r. | -0.53 | -0.07 | n.r. |
| Minematsu [40] | Muscle strength | Handgrip Strength (Female) | Partial regression coefficient | -0.114 | n.r. | -0.148 | -0.080 | n.r. |
| Minematsu [40] | Muscle strength | Handgrip Strength (Male) | Partial regression coefficient | -0.079 | n.r. | -0.104 | -0.053 | n.r. |
| Minematsu [40] | Muscle strength | Knee Extension Strength (Female) | Partial regression coefficient | -0.367 | n.r. | -0.463 | -0.272 | n.r. |
| Minematsu [40] | Muscle strength | Knee Extension Strength (Male) | Partial regression coefficient | -0.167 | n.r. | -0.247 | -0.860 | n.r. |
| Minematsu [40] | Muscle strength | Knee Flexion Strength (Female) | Partial regression coefficient | -0.671 | n.r. | -0.854 | -0.489 | n.r. |
| Minematsu [40] | Muscle strength | Knee Flexion Strength (Male) | Partial regression coefficient | -0.444 | n.r. | -0.581 | -0.306 | n.r.. |
| Olivares [43] | Muscle strength | Handgrip Strength | PCC | -0.228 | <0.001 | n.r. | n.r. | n.r. |
| Alcock [22] | Questionnaire | SF-36 Physical Component | PCC | -0.470 | <0.05 | n.r. | n.r. | n.r. |
| Balasubramanian [9] | Questionnaire | Community Balance and Mobility Scale | SRCC | -0.690 | <0.001 | n.r. | n.r. | n.r. |
| Cho [24] | Questionnaire | Elderly Physical Function Scale | SRCC | 0.495 | <0.001 | n.r. | n.r. | n.r. |
| de Vreede | Questionnaire | Assessment of Daily Activity Performance | PCC | -0.910 | <0.01 | n.r. | n.r. | n.r. |
| di Fabio [27] | Questionnaire | Fast Evaluation of Mobility, Balance, and Fear (mobility complaints) | SRCC | 0.600 | <0.05 | n.r. | n.r. | n.r. |
| di Fabio [27] | Questionnaire | Fast Evaluation of Mobility, Balance, and Fear (pain complaints) | SRCC | 0.010 | n.r. | n.r. | n.r. | n.r. |
| di Fabio [27] | Questionnaire | Fast Evaluation of Mobility, Balance, and Fear (strength complaints) | SRCC | 0.420 | <0.05 | n.r. | n.r. | n.r. |
| di Fabio [27] | Questionnaire | Fast Evaluation of Mobility, Balance, and Fear (task completion) | SRCC | -0.380 | <0.05 | n.r. | n.r. | n.r. |
| Gordt [30] | Questionnaire | Community Balance and Mobility Scale (German) | SRCC | -0.580 | n.r. | -0.74 | -0.36 | n.r. |
| Härdi [33] | Questionnaire | Continuous-Scale Physical Functional Performance Test (German) | PCC | -0.710 | <0.01 | n.r. | n.r. | n.r. |
| Hashidate [34] | Questionnaire | Life-Space Assessment | SRCC | 0.060 | 0.840 | n.r. | n.r. | n.r. |
| Hashidate [34] | Questionnaire | Life-Space Mobility at Home | SRCC | -0.740 | 0.010 | n.r. | n.r. | n.r. |
| Kwan [37] | Questionnaire | Self-Reported Health | PCC | 0.230 | <0.01 | n.r. | n.r. | n.r. |
| Kwan [37] | Questionnaire | Vitality | PCC | 0.210 | <0.001 | n.r. | n.r. | n.r. |
| Lin [38] | Questionnaire | Activities of Daily Living | n.r. Convergent | -0.450 | n.r. | n.r. | n.r. | n.r. |
| Lin [38] | Questionnaire | Activities of Daily Living (Disability) | OR | 16.500 | n.r. | n.r. | n.r. | 12 |
| Lin [38] | Questionnaire | Activities of Daily Living (No Disability) | OR | 11.200 | n.r. | n.r. | n.r. | 5 |
| Mathis [16] | Questionnaire | Patient Specific Functional Scale | PCC | -0.016 | 0.140 | n.r. | n.r. | n.r. |
| Looijaard [39] | Sarcopenia | Diagnostic Validity Sarcopenia (Baumgarter) | OR | 1.010 | n.r. | 1.00 | 1.02 | n.r. |
| Looijaard [39] | Sarcopenia | Diagnostic Validity Sarcopenia (EWGSOP) | OR | 1.000 | n.r. | 0.99 | 1.02 | n.r. |
| Looijaard [39] | Sarcopenia | Diagnostic Validity Sarcopenia (FNIH) | OR | 1.010 | n.r. | 0.99 | 1.01 | n.r. |
| Looijaard [39] | Sarcopenia | Diagnostic Validity Sarcopenia (IWGS) | OR | 1.010 | n.r. | 1.00 | 1.02 | n.r. |
| Looijaard [39] | Sarcopenia | Diagnostic Validity Sarcopenia (Janssen) | OR | 1.000 | n.r. | 0.99 | 1.01 | n.r. |
| Alcock [22] | Short walking test | 10m Gait Speed | PCC | -0.830 | <0.05 | n.r. | n.r. | n.r. |
| Hachiya [32] | Short walking test | 10m Walk Test (Female) | PCC | 0.810 | <0.01 | n.r. | n.r. | n.r. |
| Hachiya [32] | Short walking test | 10m Walk Test (Male) | PCC | 0.870 | <0.01 | n.r. | n.r. | n.r. |
| Hachiya [32] | Short walking test | 50m Walk Test (Female) | PCC | 0.960 | <0.01 | n.r. | n.r. | n.r. |
| Hachiya [32] | Short walking test | 50m Walk Test (Male) | PCC | 0.810 | <0.01 | n.r. | n.r. | n.r. |
| Lin [38] | Short walking test | 3m Walking Speed | n.r. Convergent | -0.530 | n.r. | n.r. | n.r. | n.r. |
| Lin [38] | Short walking test | Tinetti Gait | n.r. Convergent | -0.530 | n.r. | n.r. | n.r. | n.r. |
| Özden [44] | Short walking test | 50f Forward Walk (Test 1) | SRCC | 0.550 | <0.01 | n.r. | n.r. | n.r. |
| Özden [44] | Short walking test | 50f Forward Walk (Test 2) | SRCC | 0.596 | <0.01 | n.r. | n.r. | n.r. |
| Wrisley [51] | Short walking test | Functional Gait Assessment | SRCC | -0.840 | <0.001 | n.r. | n.r. | n.r. |
| Cho [24] | Long walking test | 6 Minute Walk | SRCC | -0.752 | <0.001 | n.r. | n.r. | n.r. |
| Olivares [43] | Long walking test | 6 Minute Walk | PCC | -0.573 | <0.001 | n.r. | n.r. | n.r. |
| di Fabio [27] | Rest | Fast Evaluation of Mobility, Balance, and Fear (fear complaints) | SRCC | -0.020 | n.r. | n.r. | n.r. | n.r. |
| di Fabio [27] | Rest | Fast Evaluation of Mobility, Balance, and Fear (risk factors) | SRCC | 0.370 | <0.05 | n.r. | n.r. | n.r. |
| Kwan [37] | Rest | Bodily Pain | PCC | 0.290 | <0.001 | n.r. | n.r. | n.r. |
| Kwan [37] | Rest | Fear of Falling | PCC | 0.200 | <0.01 | n.r. | n.r. | n.r. |
| Kwan [37] | Rest | Gariatric Depression Scale Score | PCC | 0.170 | <0.01 | n.r. | n.r. | n.r. |
| Kwan [37] | Rest | MMSE | PCC | -0.300 | <0.001 | n.r. | n.r. | n.r. |
| Olivares [43] | Rest | Back Scratch | PCC | 0.317 | <0.001 | n.r. | n.r. | n.r. |
| Olivares [43] | Rest | Sit and Reach | PCC | -0.292 | <0.001 | n.r. | n.r. | n.r. |

Table S 3 Construct validity of 4m gait speed test.

| First author | Comparator group | Comparator | Method | Value | P value | 95%CI lower | 95%CI upper | SD |
| --- | --- | --- | --- | --- | --- | --- | --- | --- |
| Goldberg [53] | Balance | Maximal Step Length | PCC | 0.650 | <0.01 | n.r. | n.r. | n.r. |
| Bean [10] | Muscle strength | Stair Climb Power | SRCC | 0.290 | <0.001 | n.r. | n.r. | n.r. |
| Bean [10] | Muscle strength | Double Leg Press Power (40% one rep maximum) | SRCC | 0.540 | <0.001 | n.r. | n.r. | n.r. |
| Bean [10] | Muscle strength | Double Leg Press Power (70% one rep maximum) | SRCC | 0.560 | <0.001 | n.r. | n.r. | n.r. |
| Maggio [54] | Muscle strength | Handgrip Strength (Female) | PCC | 0.380 | <0.0001 | n.r. | n.r. | n.r. |
| Maggio [54] | Muscle strength | Handgrip Strength (Male) | PCC | 0.510 | <0.0001 | n.r. | n.r. | n.r. |
| Fusco [11] | Questionnaire | Activities of Daily Living | SRCC | 0.514 | <0.001 | n.r. | n.r. | n.r. |
| Fusco [11] | Questionnaire | Instrumental Activities of Daily Living | SRCC | 0.617 | <0.001 | n.r. | n.r. | n.r. |
| Gordt [30] | Questionnaire | Community Balance and Mobility Scale (German) | SRCC | -0.580 | <0.001 | -0.74 | -0.36 | n.r. |
| Riwniak [20] | Questionnaire | Neuro-QOL Lower Extremity-Mobility Function | PCC | 0.570 | <0.05 | n.r. | n.r. | n.r. |
| Looijaard [39] | Sarcopenia | Diagnostic Validity Sarcopenia (Baumgarter) | n.r. | 0.410 | n.r. | 0.08 | 2.07 | n.r. |
| Looijaard [39] | Sarcopenia | Diagnostic Validity Sarcopenia (FNIH) | OR | 0.720 | n.r. | 0.02 | 21.40 | n.r. |
| Looijaard [39] | Sarcopenia | Diagnostic Validity Sarcopenia (Janssen) | OR | 1.540 | n.r. | 0.30 | 7.83 | n.r. |
| Fernández-Huerta [52] | Short walking test | 10m Gait Speed | ICC | 0.867 | n.r. | 0.813 | 0.905 | n.r. |
| Pasma [55] | Short walking test | 10m Gait Speed | Mean Difference | -0.110 | <0.001 | n.r. | n.r. | n.r. |
| Pasma [55] | Short walking test | 10m Gait Speed | LoA | -0.13;0.10 | n.r. | n.r. | n.r. | n.r. |
| Rolland [56] | Short walking test | 400m Walk (Baseline) | SRCC | 0.930 | n.r. | n.r. | n.r. | n.r. |
| Rolland [56] | Short walking test | 4m Gait Speed (Test 2) | PCC | 0.824 | <0.001 | n.r. | n.r. | n.r. |
| Maggio [54] | Long walking test | 6 Minute Walk (Female) | PCC | 0.490 | <0.0001 | n.r. | n.r. | n.r. |
| Maggio [54] | Long walking test | 6 Minute Walk (Male) | PCC | 0.590 | <0.0001 | n.r. | n.r. | n.r. |
| Pasma [55] | Long walking test | 6 Minute Walk | Mean Difference | -0.030 | 0.340 | n.r. | n.r. | n.r. |
| Pasma [55] | Long walking test | 6 Minute Walk | LoA | -0.08;0.03 | n.r. | n.r. | n.r. | n.r. |

Table S 4 Construct validity of 400m walk test.

| First author | Comparator group | Comparator | Method | Value | P value | 95%CI lower | 95%CI upper | SD |
| --- | --- | --- | --- | --- | --- | --- | --- | --- |
| Simonsick [58] | Balance | Standing Balance Score (Female) | PCC | -0.307 | <0.0001 | n.r. | n.r. | n.r. |
| Simonsick [58] | Balance | Standing Balance Score (Male) | PCC | -0.329 | <0.0001 | n.r. | n.r. | n.r. |
| Simonsick [58] | Balance | Walk Score (Narrow) (Female) | PCC | -0.531 | <0.0001 | n.r. | n.r. | n.r. |
| Simonsick [58] | Balance | Walk Score (Narrow) (Male) | PCC | -0.476 | <0.0001 | n.r. | n.r. | n.r. |
| Simonsick [58] | Chair stand tests | Chair Stand Test (Female) | PCC | -0.376 | <0.0001 | n.r. | n.r. | n.r. |
| Simonsick [58] | Chair stand tests | Chair Stand Test (Male) | PCC | -0.416 | <0.0001 | n.r. | n.r. | n.r. |
| Simonsick [58] | Questionnaire | Reported Ease of Walking 1 mile (Female) | PCC | -0.377 | <0.0001 | n.r. | n.r. | n.r. |
| Simonsick [58] | Questionnaire | Reported Ease of Walking 1 mile (Male) | PCC | -0.316 | <0.0001 | n.r. | n.r. | n.r. |
| Simonsick [58] | Questionnaire | Reported ease lifting 20lbs (Female) | PCC | -0.265 | <0.0001 | n.r. | n.r. | n.r. |
| Simonsick [58] | Questionnaire | Reported ease lifting 20lbs (Male) | PCC | -0.238 | <0.0001 | n.r. | n.r. | n.r. |
| Simonsick [58] | Questionnaire | Established Populations for the Epidemiologic Studies of the Elderly Scale (Female) | PCC | -0.461 | <0.0001 | n.r. | n.r. | n.r. |
| Simonsick [58] | Questionnaire | Established Populations for the Epidemiologic Studies of the Elderly Scale (Male) | PCC | -0.450 | <0.0001 | n.r. | n.r. | n.r. |
| Simonsick [58] | Questionnaire | Health ABC Performance Score (Female) | PCC | -0.614 | <0.0001 | n.r. | n.r. | n.r. |
| Simonsick [58] | Questionnaire | Health ABC Performance Score (Male) | PCC | -0.603 | <0.0001 | n.r. | n.r. | n.r. |
| Simonsick [58] | Questionnaire | Reported Ease of Walking 20 steps (Female) | PCC | -0.332 | <0.0001 | n.r. | n.r. | n.r. |
| Simonsick [58] | Questionnaire | Reported Ease of Walking 20 steps (Male) | PCC | -0.320 | <0.0001 | n.r. | n.r. | n.r. |
| Simonsick [58] | Short walking test | Walk Score 6m walk (Usual) (Female) | PCC | -0.650 | <0.0001 | n.r. | n.r. | n.r. |
| Simonsick [58] | Short walking test | Walk Score 6m walk (Usual) (Male) | PCC | -0.589 | <0.0001 | n.r. | n.r. | n.r. |
| Rolland [56] | Short walking test | 4m Gait Speed | SRCC | 0.930 | n.r. | n.r. | n.r. | n.r. |
| Simonsick [58] | Short walking test | 20m Walk Speed (Female) | PCC | -0.770 | <0.0001 | n.r. | n.r. | n.r. |
| Simonsick [58] | Short walking test | 20m Walk Speed (Male) | PCC | -0.764 | <0.0001 | n.r. | n.r. | n.r. |
| Simonsick [58] | Short walking test | 2min Walk Distance (Female) | PCC | -0.838 | <0.0001 | n.r. | n.r. | n.r. |
| Simonsick [58] | Short walking test | 2min Walk Distance (Male) | PCC | -0.828 | <0.0001 | n.r. | n.r. | n.r. |

# Appendix I: PRISMA Checklist

| Section and Topic | Item # | Checklist item | Location where item is reported |
| --- | --- | --- | --- |
| TITLE | | | |
| Title | 1 | Identify the report as a systematic review. | Title (p1) |
| ABSTRACT | | | |
| Abstract | 2 | See the PRISMA 2020 for Abstracts checklist. | Abstract (p1) |
| INTRODUCTION | | | |
| Rationale | 3 | Describe the rationale for the review in the context of existing knowledge. | Introduction (p2) |
| Objectives | 4 | Provide an explicit statement of the objective(s) or question(s) the review addresses. | Introduction (p2) |
| METHODS |  |  |  |
| *Followed guidelines* | *PC2* | *Specify, with citations, the methodology and/or guidelines used to conduct the systematic review.* | *Protocol and registration (p3)* |
| Eligibility criteria | 5 | Specify the inclusion and exclusion criteria for the review and how studies were grouped for the syntheses. | Eligibility criteria (p3) |
| Information sources | 6 | Specify all databases, registers, websites, organisations, reference lists and other sources searched or consulted to identify studies. Specify the date when each source was last searched or consulted. | Search strategy (p3) |
| Search strategy | 7 | Present the full search strategies for all databases, registers and websites, including any filters and limits used. | Supplementary file: Appendix A |
| Selection process | 8 | Specify the methods used to decide whether a study met the inclusion criteria of the review, including how many reviewers screened each record and each report retrieved, whether they worked independently, and if applicable, details of automation tools used in the process. | Search strategy (p3) |
| Data collection process | 9 | Specify the methods used to collect data from reports, including how many reviewers collected data from each report, whether they worked independently, any processes for obtaining or confirming data from study investigators, and if applicable, details of automation tools used in the process. | Data extraction (p4) |
| Data items | 10a | List and define all outcomes for which data were sought. Specify whether all results that were compatible with each outcome domain in each study were sought (e.g. for all measures, time points, analyses), and if not, the methods used to decide which results to collect. | Data extraction (p4) |
|  | 10b | List and define all other variables for which data were sought (e.g. participant and intervention characteristics, funding sources). Describe any assumptions made about any missing or unclear information. | Data extraction (p4) |
| Study risk of bias assessment | 11 | Specify the methods used to assess risk of bias in the included studies, including details of the tool(s) used, how many reviewers assessed each study and whether they worked independently, and if applicable, details of automation tools used in the process. | Methodological Quality (p4) |
| *Measurement properties* | *PC3* | *Specify the methods used to rate the results of a measurement property for each individual study and for the summarized or pooled results. Specify how many reviewers rated each study and whether they worked independently.* | *Evaluation of psychometric evidence (p4-5)* |
| Effect measures | 12 | Specify for each outcome the effect measure(s) (e.g. risk ratio, mean difference) used in the synthesis or presentation of results. | Evaluation of psychometric evidence (p4-5) |
| Synthesis methods | 13a | Describe the processes used to decide which studies were eligible for each synthesis (e.g. tabulating the study intervention characteristics and comparing against the planned groups for each synthesis (item #5)). | Evaluation of psychometric evidence (p4-5) |
|  | 13b | Describe any methods required to prepare the data for presentation or synthesis, such as handling of missing summary statistics, or data conversions. | Evaluation of psychometric evidence (p4-5) |
|  | 13c | Describe any methods used to tabulate or visually display results of individual studies and syntheses. | Evaluation of psychometric evidence (p4-5) |
|  | 13d | Describe any methods used to synthesize results and provide a rationale for the choice(s). If meta-analysis was performed, describe the model(s), method(s) to identify the presence and extent of statistical heterogeneity, and software package(s) used. | Evaluation of psychometric evidence (p4-5) |
|  | 13e | Describe any methods used to explore possible causes of heterogeneity among study results (e.g. subgroup analysis, meta-regression). | Evaluation of psychometric evidence (p4-5) |
|  | 13f | Describe any sensitivity analyses conducted to assess robustness of the synthesized results. | Evaluation of psychometric evidence (p4-5) |
| Reporting bias assessment | 14 | Describe any methods used to assess risk of bias due to missing results in a synthesis (arising from reporting biases). | Methodological Quality (p4) |
| Certainty assessment | 15 | Describe any methods used to assess certainty (or confidence) in the body of evidence for an outcome. | Evaluation of psychometric evidence (p4-5) |
| *Formulating recommendations* | *PC4* | *If appropriate, describe any methods used to formulate recommendations regarding the suitability of an OMI for a particular use.* | *Evaluation of psychometric evidence (p4-5)* |
| RESULTS | | | |
| Study selection | 16a | Describe the results of the search and selection process, from the number of records identified in the search to the number of studies included in the review, ideally using a flow diagram. | Results (p5) Figure 1 |
|  | 16b | Cite studies that might appear to meet the inclusion criteria, but which were excluded, and explain why they were excluded. | Not applicable |
| *OMI characteristics* | *PC5* | *Present characteristics of each included OMI, with appropriate citations.* | *Description of instruments (p3-4) & Summary of findings (p5-6)* |
| Study characteristics | 17 | Cite each included study and present its characteristics. | Table 1 |
| Risk of bias in studies | 18 | Present assessments of risk of bias for each included study. | Supplementary file: Appendix C |
| Results of individual studies | 19 | For all outcomes, present, for each study: (a) summary statistics for each group (where appropriate) and (b) an effect estimate and its precision (e.g. confidence/credible interval), ideally using structured tables or plots. | Tables 2-6  Supplementary file: Appendix B |
| Results of syntheses | 20a | For each synthesis, briefly summarise the characteristics and risk of bias among contributing studies. | Summary of findings (p5-6) |
|  | 20b | Present results of all statistical syntheses conducted. If meta-analysis was done, present for each the summary estimate and its precision (e.g. confidence/credible interval) and measures of statistical heterogeneity. If comparing groups, describe the direction of the effect. | Tables 2-6 |
|  | 20c | Present results of all investigations of possible causes of heterogeneity among study results. | Validity (p6) |
|  | 20d | Present results of all sensitivity analyses conducted to assess the robustness of the synthesized results. | Result synthesis only possible with summarizing, no meta-analysis. |
| Reporting biases | 21 | Present assessments of risk of bias due to missing results (arising from reporting biases) for each synthesis assessed. | Tables 2-6 |
| Certainty of evidence | 22 | Present assessments of certainty (or confidence) in the body of evidence for each outcome assessed. | Tables 2-6 |
| *Interpretability and*  *feasibility* | *PC6* | *Describe interpretability and feasibility aspects for each OMI.* | *Description of instruments (p3-4)* |
| *Recommendations* | *PC7* | *If appropriate, make recommendations for suitable OMIs for a particular use.* | *Discussion (p6-8)* |
| DISCUSSION | | | |
| Discussion | 23a | Provide a general interpretation of the results in the context of other evidence. | Discussion (p6-8) |
|  | 23b | Discuss any limitations of the evidence included in the review. | Discussion (p6-8) |
|  | 23c | Discuss any limitations of the review processes used. | Discussion (p6-8) |
|  | 23d | Discuss implications of the results for practice, policy, and future research. | Discussion (p6-8) |
| OTHER INFORMATION | | | |
| Registration and protocol | 24a | Provide registration information for the review, including register name and registration number, or state that the review was not registered. | Protocol and registration (p3) |
|  | 24b | Indicate where the review protocol can be accessed, or state that a protocol was not prepared. | Protocol and registration (p3) |
|  | 24c | Describe and explain any amendments to information provided at registration or in the protocol. | Not applicable |
| Support | 25 | Describe sources of financial or non-financial support for the review, and the role of the funders or sponsors in the review. | Other information (p8) |
| Competing interests | 26 | Declare any competing interests of review authors. | Other information (p8) |
| Availability of data, code and other materials | 27 | Report which of the following are publicly available and where they can be found: template data collection forms; data extracted from included studies; data used for all analyses; analytic code; any other materials used in the review. | Not applicable |

From*:*  Page et al. (2021) [61]

Additional items added (PC 1-7) according to Elsman et al. (2022) [62]

# Supplementary File References

1. Terwee, C.B., et al., *Development of a methodological PubMed search filter for finding studies on measurement properties of measurement instruments.* Qual Life Res, 2009. **18**(8): p. 1115-23.

2. Guralnik, J.M., et al., *A short physical performance battery assessing lower extremity function: association with self-reported disability and prediction of mortality and nursing home admission.* J Gerontol, 1994. **49**(2): p. M85-94.

3. Guralnik, J.M., et al., *Lower extremity function and subsequent disability: consistency across studies, predictive models, and value of gait speed alone compared with the short physical performance battery.* J Gerontol A Biol Sci Med Sci, 2000. **55**(4): p. M221-31.

4. Podsiadlo, D. and S. Richardson, *The timed "Up & Go": a test of basic functional mobility for frail elderly persons.* J Am Geriatr Soc, 1991. **39**(2): p. 142-8.

5. Mathias, S., U.S. Nayak, and B. Isaacs, *Balance in elderly patients: the "get-up and go" test.* Arch Phys Med Rehabil, 1986. **67**(6): p. 387-9.

6. Mokkink, L.B., et al., *COSMIN Risk of Bias tool to assess the quality of studies on reliability and measurement error of outcome measurement instrument. User Manual.* 2021.

7. Prinsen, C.A.C., et al., *COSMIN guideline for systematic reviews of patient-reported outcome measures.* Qual Life Res, 2018. **27**(5): p. 1147-1157.

8. Balachandran, A.T., et al., *Validity, reliability, and measurement error of a sit-to-stand power test in older adults: A pre-registered study.* Exp Gerontol, 2021. **145**: p. 111202.

9. Balasubramanian, C.K., *The community balance and mobility scale alleviates the ceiling effects observed in the currently used gait and balance assessments for the community-dwelling older adults.* J Geriatr Phys Ther, 2015. **38**(2): p. 78-89.

10. Bean, J.F., et al., *Is stair climb power a clinically relevant measure of leg power impairments in at-risk older adults?* Arch Phys Med Rehabil, 2007. **88**(5): p. 604-9.

11. Fusco, O., et al., *Physical function and perceived quality of life in older persons.* Aging Clin Exp Res, 2012. **24**(1): p. 68-73.

12. Gomez, J.F., et al., *Validity and reliability of the Short Physical Performance Battery (SPPB): a pilot study on mobility in the Colombian Andes.* Colomb Med (Cali), 2013. **44**(3): p. 165-71.

13. Gray, M., S. Paulson, and M. Powers, *Maximal, Not Habitual, Walking Velocity is More Highly Correlated to Functional Fitness of Community-Dwelling Older Adults.* J Aging Phys Act, 2016. **24**(2): p. 305-10.

14. Lee, S.Y., et al., *SPPB reference values and performance in assessing sarcopenia in community-dwelling Singaporeans - Yishun study.* BMC Geriatr, 2021. **21**(1): p. 213.

15. Löppönen, A., et al., *Association Between Free-Living Sit-to-Stand Transition Characteristics, and Lower-Extremity Performance, Fear of Falling, and Stair Negotiation Difficulties Among Community-Dwelling 75 to 85-Year-Old Adults.* J Gerontol A Biol Sci Med Sci, 2022. **77**(8): p. 1644-1653.

16. Mathis, R.A., et al., *Reliability and Validity of the Patient-Specific Functional Scale in Community-Dwelling Older Adults.* J Geriatr Phys Ther, 2019. **42**(3): p. E67-E72.

17. Ni, M., et al., *Reliability, Validity, and Minimal Detectable Change of Four-Step Stair Climb Power Test in Community-Dwelling Older Adults.* Phys Ther, 2017. **97**(7): p. 767-773.

18. Perera, S., et al., *Meaningful change and responsiveness in common physical performance measures in older adults.* J Am Geriatr Soc, 2006. **54**(5): p. 743-9.

19. Portegijs, E., et al., *Validity of a single question to assess habitual physical activity of community-dwelling older people.* Scand J Med Sci Sports, 2017. **27**(11): p. 1423-1430.

20. Riwniak, C., et al., *Comparison of a Multi-Component Physical Function Battery to Usual Walking Speed for Assessing Lower Extremity Function and Mobility Limitation in Older Adults.* J Nutr Health Aging, 2020. **24**(8): p. 906-913.

21. Stanziano, D.C., et al., *The modified total body rotation test: a rapid, reliable assessment of physical function in older adults.* J Am Geriatr Soc, 2010. **58**(10): p. 1965-9.

22. Alcock, L., T.D. O'Brien, and N. Vanicek, *Age-related changes in physical functioning: correlates between objective and self-reported outcomes.* Physiotherapy, 2015. **101**(2): p. 204-13.

23. Beauchamp, M.K., et al., *Reliability and Minimal Detectable Change Values for Performance-Based Measures of Physical Functioning in the Canadian Longitudinal Study on Aging.* J Gerontol A Biol Sci Med Sci, 2021. **76**(11): p. 2030-2038.

24. Cho, B.L., D. Scarpace, and N.B. Alexander, *Tests of stepping as indicators of mobility, balance, and fall risk in balance-impaired older adults.* J Am Geriatr Soc, 2004. **52**(7): p. 1168-73.

25. Creel, G.L., K.E. Light, and M.T. Thigpen, *Concurrent and construct validity of scores on the Timed Movement Battery.* Phys Ther, 2001. **81**(2): p. 789-98.

26. de Vreede, P.L., et al., *Reliability and validity of the Assessment of Daily Activity Performance (ADAP) in community-dwelling older women.* Aging Clin Exp Res, 2006. **18**(4): p. 325-33.

27. Di Fabio, R.P. and R. Seay, *Use of the "fast evaluation of mobility, balance, and fear" in elderly community dwellers: validity and reliability.* Phys Ther, 1997. **77**(9): p. 904-17.

28. Gamerman, Y., et al., *Validity and Inter-observer Reliability of the TURN 180 Test to Identify Older Adults Who Reported Falls.* Isr Med Assoc J, 2019. **21**(4): p. 269-274.

29. Goldberg, A., S. Schepens, and M. Wallace, *Concurrent validity and reliability of the maximum step length test in older adults.* J Geriatr Phys Ther, 2010. **33**(3): p. 122-7.

30. Gordt, K., et al., *German version of the Community Balance and Mobility Scale : Translation and evaluation of measurement properties.* Z Gerontol Geriatr, 2019. **52**(1): p. 28-36.

31. Griswold, D., et al., *Establishing the reliability and concurrent validity of physical performance tests using virtual reality equipment for community-dwelling healthy elders.* Disabil Rehabil, 2015. **37**(12): p. 1097-101.

32. Hachiya, M., et al., *Reproducibility and validity of the 50-meter walking test in community-dwelling elderly.* J Phys Ther Sci, 2015. **27**(5): p. 1511-4.

33. Härdi, I., et al., *Validity of the German Version of the Continuous-Scale Physical Functional Performance 10 Test.* J Aging Res, 2017. **2017**: p. 9575214.

34. Hashidate, H., et al., *Measuring indoor life-space mobility at home in older adults with difficulty to perform outdoor activities.* J Geriatr Phys Ther, 2013. **36**(3): p. 109-14.

35. Kim, M.J., et al., *Validation of lower extremity performance tests for determining the mobility limitation levels in community-dwelling older women.* Aging Clin Exp Res, 2009. **21**(6): p. 437-44.

36. Kristensen, M.T., et al., *Interrater reliability of the standardized Timed Up and Go Test when used in hospitalized and community-dwelling older individuals.* Physiother Res Int, 2019. **24**(2): p. e1769.

37. Kwan, M.M., et al., *Sensorimotor function, balance abilities and pain influence Timed Up and Go performance in older community-living people.* Aging Clin Exp Res, 2011. **23**(3): p. 196-201.

38. Lin, M.R., et al., *Psychometric comparisons of the timed up and go, one-leg stand, functional reach, and Tinetti balance measures in community-dwelling older people.* J Am Geriatr Soc, 2004. **52**(8): p. 1343-8.

39. Looijaard, S., et al., *Single Physical Performance Measures Cannot Identify Geriatric Outpatients with Sarcopenia.* J Frailty Aging, 2018. **7**(4): p. 262-267.

40. Minematsu, A., et al., *Association between muscle strength and physical performance in Japanese elderly: The Fujiwara-kyo Study.* Journal of Clinical Gerontology & Geriatrics, 2018. **9**(2): p. 44-51.

41. Nepal, G.M., M. Basaula, and S. Sharma, *Inter-rater reliability of Timed Up and Go test in older adults measured by physiotherapist and caregivers.* European Journal of Physiotherapy, 2020. **22**(6): p. 325-331.

42. O'Hoski, S., et al., *Construct validity of the BESTest, mini-BESTest and briefBESTest in adults aged 50 years and older.* Gait Posture, 2015. **42**(3): p. 301-5.

43. Olivares, P.R., et al., *Fitness and health-related quality of life dimensions in community-dwelling middle aged and older adults.* Health Qual Life Outcomes, 2011. **9**: p. 117.

44. Özden, F., et al., *The test-retest reliability and concurrent validity of the 3-m backward walk test and 50-ft walk test in community-dwelling older adults.* Ir J Med Sci, 2022. **191**(2): p. 921-928.

45. Schaubert, K.L. and R.W. Bohannon, *Reliability and validity of three strength measures obtained from community-dwelling elderly persons.* J Strength Cond Res, 2005. **19**(3): p. 717-20.

46. Schepens, S., A. Goldberg, and M. Wallace, *The short version of the Activities-specific Balance Confidence (ABC) scale: its validity, reliability, and relationship to balance impairment and falls in older adults.* Arch Gerontol Geriatr, 2010. **51**(1): p. 9-12.

47. Steffen, T.M., T.A. Hacker, and L. Mollinger, *Age- and gender-related test performance in community-dwelling elderly people: Six-Minute Walk Test, Berg Balance Scale, Timed Up & Go Test, and gait speeds.* Phys Ther, 2002. **82**(2): p. 128-37.

48. Suwannarat, P., et al., *The use of functional performance tests by primary health-care providers to determine walking ability with and without awalking device in community-dwelling elderly.* Physiother Theory Pract, 2021. **37**(1): p. 64-72.

49. Suzuki, Y., et al., *Absolute reliability of measurements of muscle strength and physical performance measures in older people with high functional capacities.* Eur Geriatr Med, 2019. **10**(5): p. 733-740.

50. Wang, C.Y., et al., *Psychometric properties of the Berg Balance Scale in a community-dwelling elderly resident population in Taiwan.* J Formos Med Assoc, 2006. **105**(12): p. 992-1000.

51. Wrisley, D.M. and N.A. Kumar, *Functional gait assessment: concurrent, discriminative, and predictive validity in community-dwelling older adults.* Phys Ther, 2010. **90**(5): p. 761-73.

52. Fernandez-Huerta, L. and K. Cordova-Leon, *Reliability of two gait speed tests of different timed phases and equal non-timed phases in community-dwelling older persons.* Medwave, 2019. **19**(3): p. e7611.

53. Goldberg, A. and S. Schepens, *Measurement error and minimum detectable change in 4-meter gait speed in older adults.* Aging Clin Exp Res, 2011. **23**(5-6): p. 406-12.

54. Maggio, M., et al., *Instrumental and Non-Instrumental Evaluation of 4-Meter Walking Speed in Older Individuals.* PLoS One, 2016. **11**(4): p. e0153583.

55. Pasma, J.H., et al., *Walking speed in elderly outpatients depends on the assessment method.* Age (Dordr), 2014. **36**(6): p. 9736.

56. Rolland, Y.M., et al., *Reliability of the 400-m usual-pace walk test as an assessment of mobility limitation in older adults.* J Am Geriatr Soc, 2004. **52**(6): p. 972-6.

57. Van Ancum, J.M., et al., *Gait speed assessed by a 4-m walk test is not representative of daily-life gait speed in community-dwelling adults.* Maturitas, 2019. **121**: p. 28-34.

58. Simonsick, E.M., et al., *Measuring higher level physical function in well-functioning older adults: expanding familiar approaches in the Health ABC study.* J Gerontol A Biol Sci Med Sci, 2001. **56**(10): p. M644-9.

59. Mokkink, L.B., et al., *COSMIN Risk of Bias tool to assess the quality of studies on reliability or measurement error of outcome measurement instruments: a Delphi study.* BMC Med Res Methodol, 2020. **20**(1): p. 293.

60. Mokkink, L.B., et al., *COSMIN Risk of Bias checklist for systematic reviews of Patient-Reported Outcome Measures.* Qual Life Res, 2018. **27**(5): p. 1171-1179.

61. Page, M.J., et al., *The PRISMA 2020 statement: an updated guideline for reporting systematic reviews.* BMJ, 2021. **372**: p. n71.

62. Elsman, E.B.M., et al., *Study protocol for developing, piloting and disseminating the PRISMA-COSMIN guideline: a new reporting guideline for systematic reviews of outcome measurement instruments.* Systematic Reviews, 2022. **11**(1): p. 121.
